# Halide Perovskite Heterostructures for High-Performance Light-Emitting Diodes

**DOI:** 10.1007/s40820-025-02038-y

**Published:** 2026-01-05

**Authors:** Yiming Huo, Tingwei He, Shaopeng Yang, Yuanzhi Jiang, Changjiu Sun

**Affiliations:** 1 Province-Ministry Co-Construction Collaborative Innovation Center of Hebei Photovoltaic Technology, Hebei Key Laboratory of Optic-Electronic Information and Materials, College of Physics Science and Technology, Hebei University, Baoding, 071002 Hebei People’s Republic of China; 2 State Key Laboratory of Advanced Chemical Power Sources, Frontiers Science Center for New Organic Matter, Key Laboratory of Advanced Energy Materials Chemistry (Ministry of Education), College of Chemistry, Nankai University, Tianjin, 300071 People’s Republic of China

**Keywords:** Halide perovskite, Heterostructure, Electroluminescence, Perovskite light-emitting diode

## Abstract

Metal halide perovskites have emerged as highly promising candidates for the emissive layer in next-generation light-emitting diodes (LEDs) due to their narrow emission linewidths, high photoluminescence quantum yields, and tunable emission wavelengths. Achieving high-performance perovskite LEDs (PeLEDs) requires the emissive layer to possess efficient radiative recombination, low defect density, minimal ion mobility, and effective carrier confinement. Perovskite/perovskite heterostructure (PPHS) offers a compelling approach for engineering emissive layers with these desired attributes, owing to their ability to passivate surface defects, tailor bandgaps, and suppress ion migration. PeLEDs based on PPHS have demonstrated superior performance compared to single-phase devices, particularly in terms of external quantum efficiency and operational stability. This review provides a comprehensive overview of the typical PPHS architectures applied in PeLEDs, including vertical, lateral, and bulk configurations. We discuss representative fabrication strategies and the associated optoelectronic properties of these heterostructures, highlighting the mechanisms by which they enhance device efficiency and stability. Finally, we explore the remaining challenges and prospects for the application of PPHS in PeLEDs and other luminescent technologies.

## Highlights

- This review systematically summarizes the application of perovskite/perovskite heterostructures (PPHSs) in light-emitting diodes (LEDs), highlighting their critical roles in defect passivation, carrier confinement, lattice stabilization, and light management.
- This review categorized PPHSs by dimensional combinations (e.g., 3D/3D, 2D/3D, 0D/3D) and spatial architectures—vertical, lateral, and bulk heterostructures—elucidating the structure-property relationships for efficient LEDs.
- Key challenges and future directions are outlined, including advances in high-resolution characterization, carrier dynamics analysis, and controlled synthesis of PPHSs for next-generation optoelectronic applications.

## Graphical abstract

**Figure:**
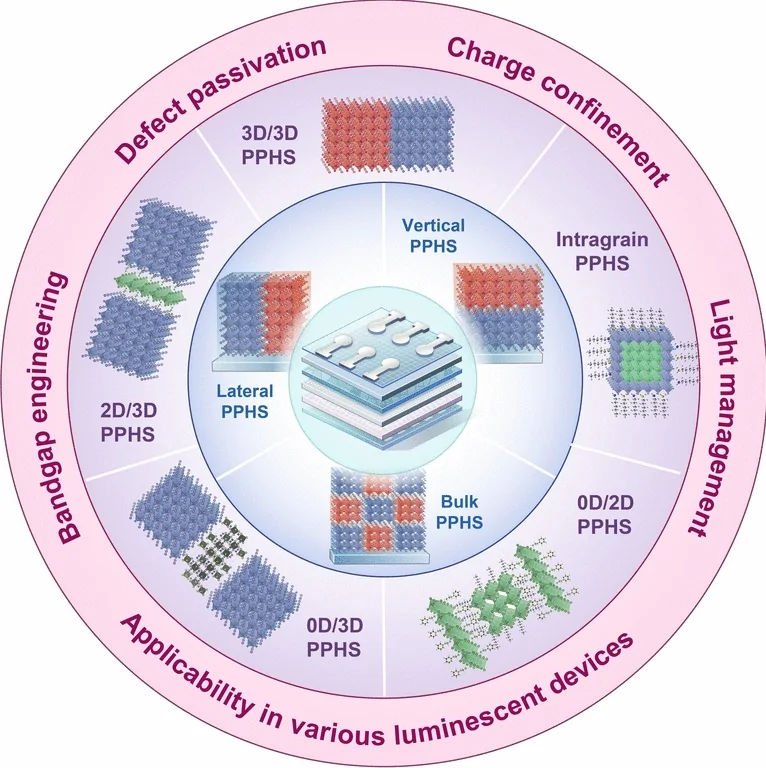

## Introduction

Metal halide perovskites have emerged as a highly attractive class of semiconductor materials, garnering significant attention in both academia and industry due to their outstanding electrical and optical properties [1–4]. These materials exhibit exceptional defect tolerance, and with appropriate defect passivation strategies, their photoluminescence quantum yield (PLQY) can be enhanced to near unity [5, 6]. Moreover, their intrinsically narrow emission linewidths (full width at half maximum ≤ 15 nm) enable ultra-high color purity, achieving color gamuts exceeding 140% of the National Television Standards Committee (NTSC) standard [7, 8]. Additionally, their compatibility with low-temperature solution processing enables cost-effective fabrication and supports large-area, continuous coating—an advantage for scalable manufacturing [9, 10]. To date, perovskite light-emitting diodes (PeLEDs) have achieved external quantum efficiencies (EQEs) exceeding 30% and operational half-lifetimes (*T*_50_) of up to 180,000 h at an initial luminance of 100 cd m^−2^, rivaling the performance of state-of-the-art commercial organic LEDs [11–14]. As such, PeLEDs are widely regarded as one of the most promising candidates for next-generation lighting and display technologies.

Metal halide perovskites offer remarkable compositional and structural tunability. The metal halide perovskites used in solar cells and LEDs have been focused on the ABX_3_ structure, where A is a monovalent cation, B is Pb or Sn, and X is a halide. Beyond the ABX_3_ structure, perovskite materials with different dimensions and chemical compositions, such as zero-dimensional A_4_BX_6_, two-dimensional AB_2_X_5,_ and copper iodide perovskites (e.g., CsCu_2_I_3_), exhibit unique chemical and physical properties and are gradually attracting the interest of researchers. A heterostructure is a composite system composed of two or more distinct semiconductor materials. Optimizing heterostructure design has become a key strategy in advancing semiconductor device performance. A well-engineered heterostructure can modulate the balance between excitons and free carriers—enhancing electron extraction in solar cells and photodetectors or, alternatively, promoting radiative recombination through spatial confinement of charge carriers, thereby improving the quantum efficiency of light-emitting diodes (LEDs). In perovskite-based optoelectronic devices, heterostructures can be broadly categorized into perovskite/other semiconductor heterojunctions and perovskite/perovskite heterojunctions (PPHSs) [15, 16]. The former includes combinations of perovskites with two-dimensional layered materials (e.g., graphene, transition metal dichalcogenides) [17–19], metal oxides (e.g., SnO_2_, ZnO, TiO_2_, NiO) [20–22], and other semiconductors, which have demonstrated enhanced optoelectronic properties suitable for a wide range of applications. Compared to these, PPHSs offer additional advantages, including greater flexibility in tuning carrier transport and recombination dynamics, reduced fabrication complexity, and lower production costs—factors that have increasingly drawn the interest of researchers [16, 23–25].

PPHSs have been shown to improve charge carrier mobility [26, 27], suppress electron–phonon coupling [28], enhance radiative recombination rates [29, 30], and improve the chemical stability of perovskite materials [31], thereby offering significant potential for high-performance PeLED development. For example, forming a type-I band alignment between two perovskite phases enables efficient charge funneling from the wide-bandgap phase into the narrow-bandgap phase, thus boosting radiative recombination [29]. Moreover, PPHS can effectively passivate surface defects that act as non-radiative recombination centers and suppress ion migration during operation, thereby enhancing device stability [32]. Furthermore, the phonon confinement effect in perovskite heterojunctions has been demonstrated to reduce the electron–phonon coupling dominated by the Frohlich interaction, thereby decreasing the FWHM of the PL spectra [28]. Overall, constructing PPHS represents a highly effective strategy for realizing high-efficiency, high-stability PeLEDs and provides a promising pathway to address the limitations of single-phase perovskite devices.

In this review, we summarize several representative PPHSs employed in PeLED devices. We classify PPHS into three types: vertical, lateral, and bulk, based on the spatial distribution of the perovskite components. On this basis, we introduced the common dimensional structures in these PPHSs, such as 3D/3D, 2D/3D, 0D/3D, and 0D/2D structures, and detailed their representative fabrication methods and optoelectronic properties. The underlying mechanisms by which these heterostructures enhance the efficiency and operational stability of PeLEDs are also discussed. Finally, we outline potential strategies for achieving precise synthesis and high-resolution characterization of PPHSs, to further improve device performance and enable broader applicability across diverse fields. We also propose future research directions to expand the use of PPHS in emerging optoelectronic applications, such as lasers and spin-polarized LEDs.

## Structure and Carrier Dynamics of PPHSs

### Structure of PPHSs

**Fig. 1:**
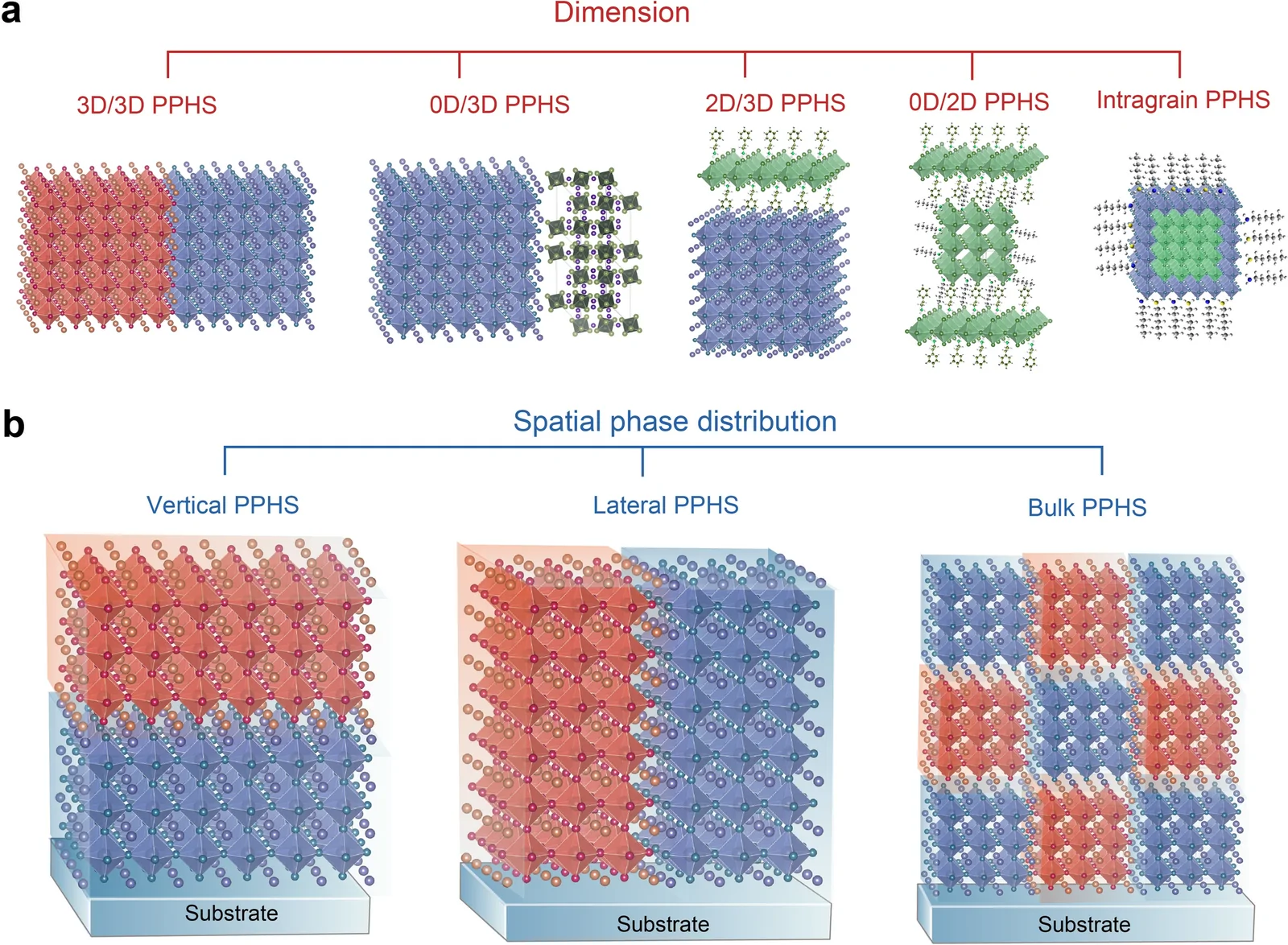
The structure of PPHSs. **a** PPHSs with 3D/3D, 0D/3D, 2D/3D, and 0D/2D configurations. **b** PPHSs with vertical, lateral, and bulk spatial phase distribution

The structure of PPHSs can be classified based on dimension and spatial phase distribution. From the perspective of dimension, PPHSs can be classified into 3D/3D, 0D/3D, 2D/3D, and 0D/2D configurations, etc., depending on the size and geometry of the perovskite grains (Fig. 1a). 3D perovskites have a fully connected octahedral framework extending in all three dimensions, and the grain size is significantly larger than the exciton Bohr radius [33]. 2D perovskite refers to nanosheets with growth restricted in the out-of-plane direction, encompassing both pure 2D perovskites with only one inorganic layer in the in-plane direction and quasi-2D perovskites with multiple inorganic layers [34, 35]. 1D PPHS refers to perovskite heterojunctions with nanowires/rods growing along a single direction. Morphological 0D perovskites refer to perovskite nanocrystals or quantum dots (QDs) with all dimension nanoscale close to or less than the excitons’ Bohr diameter. Furthermore, from a crystal structure perspective, it is worth noting that 0D perovskites also encompass A_4_PbX_6_ perovskites featuring spatially disconnected lattices. In this class of materials, the [PbX_6_]^4−^ octahedra are completely separated in space, meaning that the size of the nanocrystals does not significantly influence the band structure [36]. In these low-dimensional perovskites, the electronic structure, optoelectronic properties, and stability of perovskites undergo significant changes due to size, surface, and quantum confinement effects. The combination of these low-dimensional and 3D perovskites will exhibit superior properties such as enhanced optoelectronic performance and improved stability, driving further improvements in optoelectronic device performance.

According to their spatial arrangement, PPHSs are typically categorized as vertical, lateral, or bulk heterostructures (Fig. 1b). In vertical PPHSs, two different perovskite semiconductors are stacked perpendicularly across the film thickness. Vertical heterojunctions offer precise interface control, flexible bandgap tuning, and three-dimensional stacking capabilities, making them advantageous for constructing efficient, highly integrated optoelectronic devices. Vertical PPHSs are typically fabricated using two-step methods, where after depositing the first perovskite layer, parallel-stacked multilayer perovskites are obtained via liquid-phase epitaxy (LPE), chemical vapor deposition (CVD), spin coating, or mechanical transfer techniques. Lateral PPHSs involve planar heterojunctions formed along the single layer. Theoretically, compared to vertical heterojunctions, the lateral transport of charge carriers in lateral heterojunctions is restricted, which prevents lateral diffusion and separation of charge carriers, making them easier to recombine. This gives them an advantage in constructing high-efficiency LED devices. Lateral heterojunctions can be obtained through solution-phase growth. For example, leveraging the lateral growth characteristics of perovskite materials, a second perovskite phase can sequentially grow along the pre-generated perovskite phase on a single substrate. The bulk PPHS defined in this review refers to two or more distinct perovskite crystal phases uniformly distributed and interpenetrating in three-dimensional space, forming numerous embedded heterogeneous interfaces. These phases coexist thermodynamically or kinetically while retaining their distinct crystal structures and optoelectronic properties. Since the interfaces in bulk heterostructures are distributed throughout the entire active layer, the device exhibits higher exciton dissociation efficiency and reduced exciton recombination probability [37]. Thus, the uniform distribution of the two perovskite materials will facilitate more efficient carrier localization and minimize carrier diffusion and separation. Compared to laterally and vertically distributed PPHSs, the fabrication process for bulk PPHSs is simpler. It can be obtained through one-step processes such as spin coating, thermal evaporation, and solution-based self-assembly, or by introducing pre-synthesized perovskite materials into the crystallization process of the second phase. Nevertheless, bulk PPHSs also face the issue of low carrier transport efficiency due to random networks, imposing higher demands on the electrical properties of the materials.

### Interface of PPHSs

**Fig. 2:**
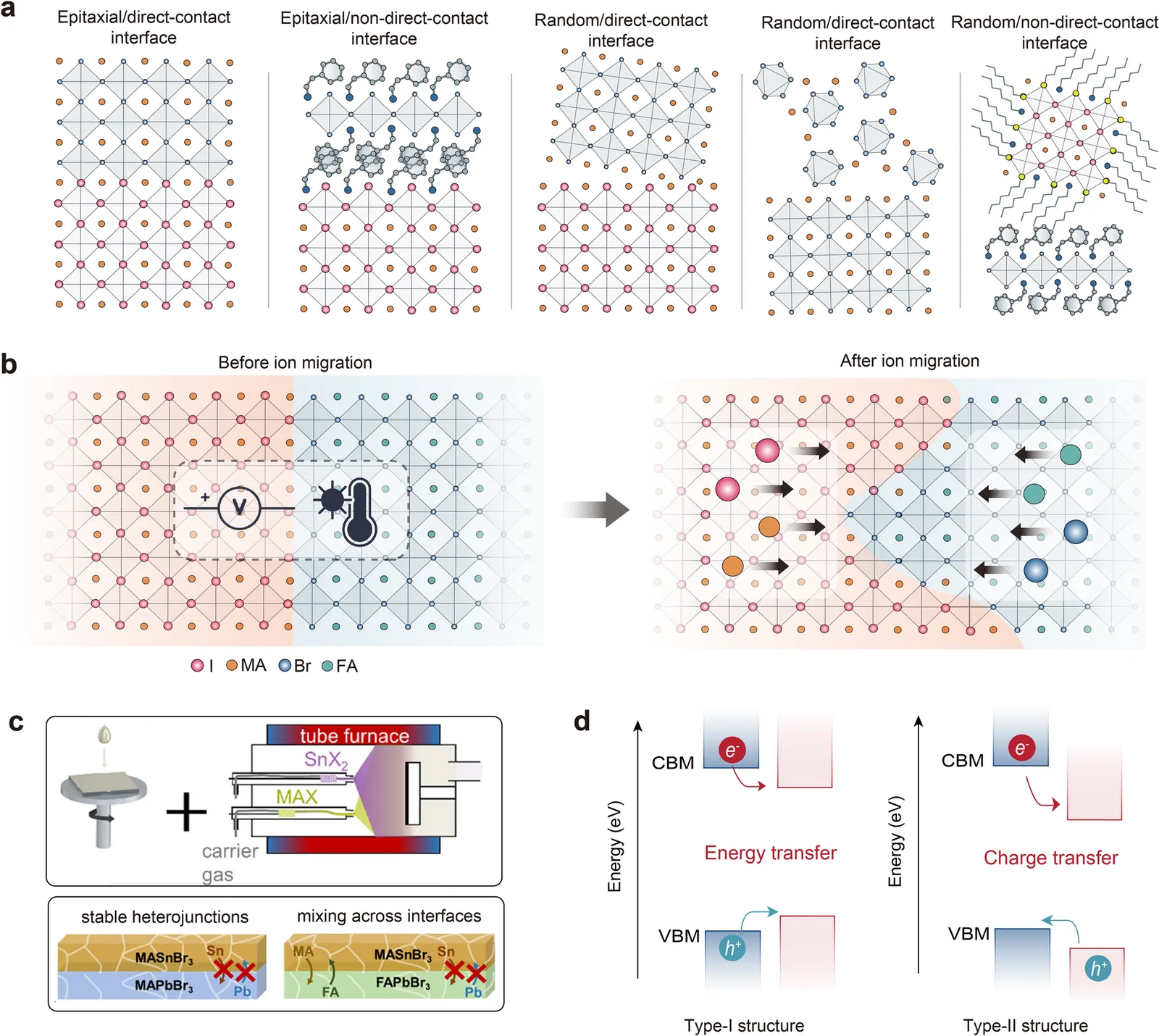
Heterojunction interface and energy-level structure of PPHSs. **a** Schematic illustration of the heterojunction interface in different PPHSs. **b** Schematic diagrams showing the ion migration in PPHSs. **c** Schematic diagrams of the PPHSs fabricated via spin coating and vapor deposition, bottom showing the ion migration trend in MA/FAPbBr_3_ and MASnBr_3_ heterojunction [40]. Copyright 2020, American Chemical Society. **d** Schematic diagrams of type-I and type-II band alignments and the corresponding charge/energy transfer process

The heterojunction interface critically affects the optoelectronic performance and structural stability of PPHSs. As shown in Fig. 2a, heterojunctions in PPHSs can be classified into epitaxial interfaces and non-epitaxial interfaces, depending on whether crystallographic coordination exists at the two-phase interface. Similarly, depending on whether an interlayer or ligands exists between the two phases, they can be classified as direct-contact or non-direct-contact interfaces. The nature of the interface directly impacts the optical and electrical properties of the heterojunction. Theoretically, epitaxially aligned, direct-contact interfaces enable the most efficient energy transfer and carrier transport [38], which are essential for achieving high-efficiency, high-brightness perovskite light-emitting diodes (PeLEDs). However, such ideal interfaces require a high degree of lattice matching; generally, a lattice mismatch exceeding 5% is considered too large to support epitaxial growth. The lattice mismatch between the two perovskite phases constituting the heterojunction also induces elastic strain fields near the boundary. Specifically, when one perovskite is epitaxially grown onto another, the material with the smaller lattice constant is “stretched,” while the material with the larger lattice constant is “compressed” to achieve atomic bonding at the interface. These interfacial strains have been shown to further influence the optical properties and stability of PPHSs.

Another key challenge is mitigating ion migration at the two-phase interface. Ion migration can blur the heterojunction boundary, resulting in the formation of alloy gradient layers (Fig. 2b). While mild ion diffusion may alleviate lattice mismatch and facilitate epitaxial growth between perovskite phases, excessive ion migration degrades spectral purity, shifts emission peaks, and can even lead to heterojunction collapse. Ion migration and mixed-phase formation are thermodynamically favorable; this process is accelerated under heat, illumination, or applied electric fields [39]. For example, in vapor-deposited perovskite multilayer films, I/Br heterojunctions undergo substantial ion exchange within an hour at 60 °C [40]. In contrast, FA/MA heterojunctions with a single halide component form a uniform mixed phase after 937 h. Pb/Sn heterojunctions with identical A-site and halide compositions retain their structural integrity even after 1,500 h, suggesting negligible B-site diffusion (Fig. 2c). This trend aligns with previously reported migration activation energy for different ions [41, 42]. The faster migration of halide ions can be attributed to their smaller ionic radii, weaker Coulombic interactions with neighboring ions, and the angle-sharing octahedral lattice configuration that provides continuous migration pathways. The ion migration rate is also influenced by crystal structure and orientation. Variations in A-site composition can induce different degrees of lattice distortion, thereby affecting halide ion mobility [43]. Additionally, the migration behavior depends on the crystal plane orientation of the heterojunction. For instance, in FA_0.875_Cs_0.125_PbBr_3_/FA_0.875_Cs_0.125_PbI_3_ heterojunctions, halide ion migration is suppressed along the (100) plane due to higher interfacial strain compared to the (010) direction [44]. This arises because the compressive strain on the (100) plane (− 2.5%) exceeds that on the (010) plane (− 0.8%) in the heterojunction. As previously reported, compressive strain not only suppresses halide vacancy formation but also slows ion migration within the perovskite, thereby enhancing material stability [45].

To maintain the band alignment and charge carrier confinement, developing stable heterostructures and mitigating ion migration at PPHS interfaces are essential. Introducing 2D materials such as graphene as ion barrier layers between heterointerfaces has been shown to maintain atomic-level interface clarity [46]. Moreover, certain heterojunctions—such as the 0D/3D Cs_4_PbCl_6_/CsPbBr_3_ combination—exhibit inherently limited anion intermixing [47]. Overall, atomic-level sharp interfaces lie in their precise band alignment, enabling accurate carrier and exciton management. In type-I structures, this alignment generates strong carrier/exciton confinement, significantly enhancing radiative recombination probability, making them ideal for high-efficiency light-emitting devices. Additionally, atomically sharp interfaces deliver higher color purity and superior spectral stability. However, significant lattice mismatch between the two materials can induce substantial local strain at the sharp interface. Excessive strain may generate defects at the interface, creating non-radiative recombination centers. Mildly graded interfaces mitigate interfacial strain, relieve lattice mismatch, and reduce defect density at the interface. However, gradient interfaces exhibit blurred band alignment, hindering effective confinement of carriers or excitons and impeding the achievement of high color purity and high radiative recombination rates. For PeLEDs pursuing high efficiency and spectral stability, atomically sharp interfaces remain the preferred choice.

### Electronic Structure and Carrier Dynamics of PPHSs

In PPHSs, the two constituent perovskite phases typically exhibit different bandgap widths and band levels, resulting in a step-like discontinuity in their electronic energy levels [48]. Based on the relative alignment of the conduction band minimum (CBM) and valence band maximum (VBM) levels, PPHS can be categorized into two primary types: type-I (straddling) and type-II (staggered) heterojunctions (Fig. 2d) [15]. In a type-I configuration, the VBM of the narrow-bandgap phase lies above that of the wide-bandgap phase, while the CBM lies below, meaning the entire bandgap of the narrow-bandgap material is nested within that of the wide-bandgap material. This configuration favors energy transfer, where high-energy carriers (typically excitons) generated in the wide-bandgap phase transfer into and recombine within the narrow-bandgap phase, enhancing radiative recombination and PLQY. Consequently, type-I PPHSs are well-suited for applications requiring efficient carrier recombination, such as high-performance PeLEDs, low-threshold lasers, and other light-emitting devices.

In contrast, type-II heterojunctions feature bandgaps that partially overlap, such that both the VBM and CBM of one material are either higher or lower than those of the other. This configuration promotes charge separation, with electrons transferring from the higher-energy CBM to the lower-energy CBM and holes moving from the deeper VBM to the shallower VBM [49]. The resulting opposite-direction carrier flows facilitate net charge separation while suppressing radiative recombination. As a result, type-II structures are more suitable for applications like photocatalysis, photodetectors, and photovoltaics, where efficient charge separation is desirable. In addition, further reducing the bandgap overlap between the two semiconductor materials and arranging them in a completely staggered pattern forms a type-III heterojunction [50]. However, this band arrangement is not conducive to charge and energy transfer and is rarely reported in perovskite devices.

Energy transfer between the two phases in PPHS can occur via two mechanisms: non-radiative and radiative transfer. Non-radiative energy transfer includes Förster resonance energy transfer (FRET) and Dexter energy transfer [51]. FRET is based on dipole–dipole coupling and is effective over distances of less than 10 nm, while Dexter transfer relies on electron exchange interactions and operates only over atomic-scale distances (≤ 1 nm). These non-radiative pathways do not involve photon emission or reabsorption, thus maintaining high energy transfer efficiency. Among these, FRET is generally considered the dominant mechanism in type-I PPHS. Because FRET depends on both the spatial proximity and dipole alignment between donor and acceptor, the relative orientation of the two perovskite phases significantly influences transfer efficiency. For example, in highly oriented heterojunction films, energy transfer from high-bandgap to low-bandgap domains is more effective than in films with randomly oriented grains [52]. Radiative energy transfer, which involves photon emission by one component and subsequent absorption by another, can operate over longer distances [53]. However, its efficiency depends on the spectral overlap between emission and absorption and the PLQY of the materials. While radiative transfer can occur in both type-I and type-II structures, in type-II configurations, interfacial charge transfer often quenches excitonic emission, reducing the efficiency of radiative energy transfer. This suggests a competitive relationship between charge and energy transfer in type-II PPHS.

Both energy and charge transfer processes in PPHSs occur on ultrafast timescales—from sub-picoseconds to several picoseconds—significantly faster than typical radiative recombination. These dynamics can be probed through time-resolved photoluminescence (TRPL) and transient absorption (TA) spectroscopy [54]. In TRPL measurements, type-II structures exhibit markedly shorter PL lifetimes than type-I structures due to rapid exciton dissociation via charge transfer. TA spectra also provide complementary insights: In energy transfer, the donor phase shows a reduced recombination lifetime, while the acceptor phase exhibits a prolonged lifetime. For charge transfer, TA measurements reveal a rapid decay of the donor signal (on the picosecond scale, indicating fast charge extraction) and a slower decay in the acceptor phase (on the nanosecond scale, corresponding to slower recombination of free carriers) [55].

## PPHSs for PeLEDs

### 3D/3D PPHSs

3D/3D PPHSs primarily fall into vertical and bulk heterojunctions. Through interface engineering, bandgap tuning, and phase design, 3D/3D PPHSs demonstrate significant potential in enhancing the efficiency and stability of PeLEDs while enabling multicolor/white light emission. Vertical heterojunctions are typically constructed using liquid-phase epitaxy or spin coating. They offer advantages in delivering high-quality single-crystal films and regulating interfacial carrier dynamics. In addition, precise control of crystallization conditions or thermal treatment processes enables the stable coexistence of two or more perovskite phases with distinct luminescent properties within the active layer, facilitating the construction of bulk heterojunctions for white light emission.

#### Vertical Structure

**Fig. 3:**
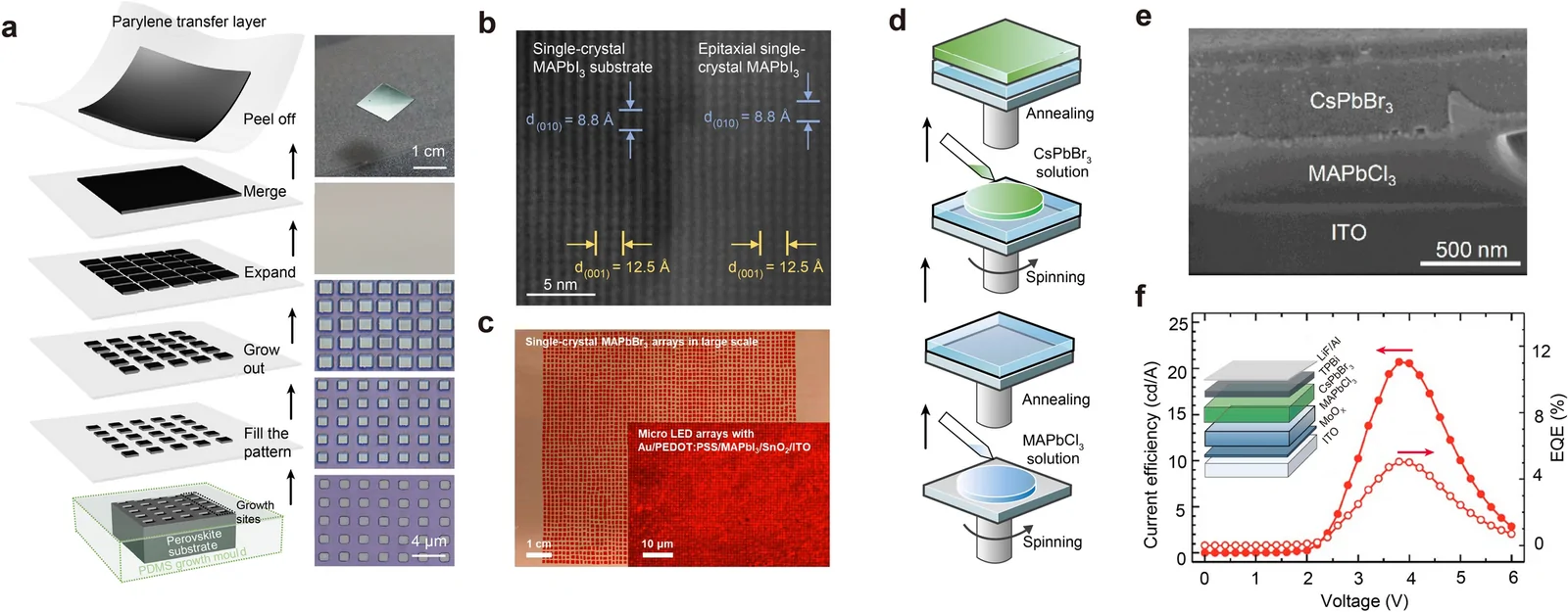
Vertical 3D/3D PPHSs.** a** Schematics and corresponding optical images showing the lithography-assisted epitaxial growth and transfer process of the single-crystal perovskite film [57]. Copyright 2020, Springer Nature. **b** Transmission electron microscope (TEM) image of the MAPbI_3_/MAPbI_3_ 3D/3D PPHS with well-aligned lattice structures [57]. Copyright 2020, Springer Nature. **c** Electroluminescence (EL) image of the transferred single-crystal MAPbBr_3_ arrays and micro-LED [57]. Copyright 2020, Springer Nature. **d** Schematic showing the dynamic spin-coating fabrication procedure for the CsPbBr_3_/MAPbCl_3_ 3D/3D PPHS. **e** Focused ion beam scanning electron microscopy (FIB-SEM) images of the CsPbBr_3_/MAPbCl_3_ double-layer structure [26]. Copyright 2020, American Chemical Society. **f** Current efficiency–EQE curves of the PeLEDs based on CsPbBr_3_ single layer and the CsPbBr_3_/MAPbCl_3_ double layer; the inset shows the device structure of the PeLED. Reproduced with permission [26]. Copyright 2020, American Chemical Society

Large-sized vertical 3D/3D single-crystal PPHSs are typically fabricated via liquid-phase epitaxy. Shewmon et al. achieved the growth of MAPbI_3_ along the surface of pre-prepared large-sized MAPbBr_3_ by immersing a MAPbBr_3_ single crystal in a saturated solution of the MAPbI_3_ precursor, resulting in centimeter-scale MAPbBr_3_/MAPbI_3_ heterostructures [56]. A typical characteristic of 3D/3D single-crystal PPHSs is that these materials lack a clear heterojunction interface. Since epitaxial growth is performed in a polar solvent, the polar solvent inevitably erodes the surface of the pre-synthesized perovskite crystals, resulting in an uneven interface boundary. The dissolution–reconstruction process of the crystal surface results in the formation of a transition interface layer containing a mixed halide region. To some extent, this transition interface reduces the lattice mismatch between different perovskite phases, enabling heteroepitaxial growth. However, bulk single-crystal heterostructures are not suitable for constructing thin-film devices. This solution-grown epitaxial MAPbBr_3_/MAPbI_3_ heterojunction perovskite single crystal is applied in photodetectors. Due to its “quasi-type-II” band alignment providing asymmetric carrier confinement, electrons are confined within the narrow-bandgap phase, while holes readily distribute across phases, promoting light-induced exciton dissociation and facilitating carrier transport. Lei et al. reported a photolithography-assisted liquid-phase epitaxial growth process for the synthesis of vertical thin single-crystal PPHSs (Fig. 3a, b) [57]. They used bulk MAPbX_3_ (X=I, Br) crystals as substrates and a 2-μm patterned polymer as a growth mask for the epitaxial growth of different single-crystal perovskites. They achieved epitaxial growth of MAPbI_3_ and MAPb_0.5_Sn_0.5_I_3_ single crystals on the substrate with thicknesses ranging from 600 nm to 100 μm, and a large area of up to 5.5 × 5.5 cm^2^. These epitaxial single-crystal films can be integrated onto different substrates via layer transfer processes, maintaining high crystallinity and strong substrate adhesion. Using this liquid-phase growth and transfer method, they demonstrated single-crystal PeLEDs with pixel sizes ranging from 1 to 100 μm, demonstrating their potential for flexible, high-resolution, and high-stability displays (Fig. 3c).

The spin-coating method is also used to produce vertical 3D/3D PPHSs. Kang et al. demonstrated the conformal deposition of a 350-nm CsPbBr_3_ layer on a 300-nm MAPbCl_3_ underlayer using a dynamic spin-coating process (Fig. 3d, e) [26]. Their work demonstrates that the MAPbCl_3_ layer enhances PL intensity and improves the stability of the CsPbBr_3_ film. This is attributed to the passivation of surface defects and the interface strain in the bilayer heterostructure, which increases the activation energy for ion migration. Additionally, the underlying MAPbCl_3_ layer in the bilayer structure can serve as a hole transport layer in LED devices. This MAPbCl_3_ hole transport layer exhibits lower leakage current and more efficient hole injection compared to the traditional PEDOT:PSS layer. PeLED based on the MAPbCl_3_/CsPbBr_3_ heterojunction demonstrates a twofold increase in efficiency compared to a traditional PEDOT:PSS-based device (Fig. 3f).

#### Bulk Structure

**Fig. 4:**
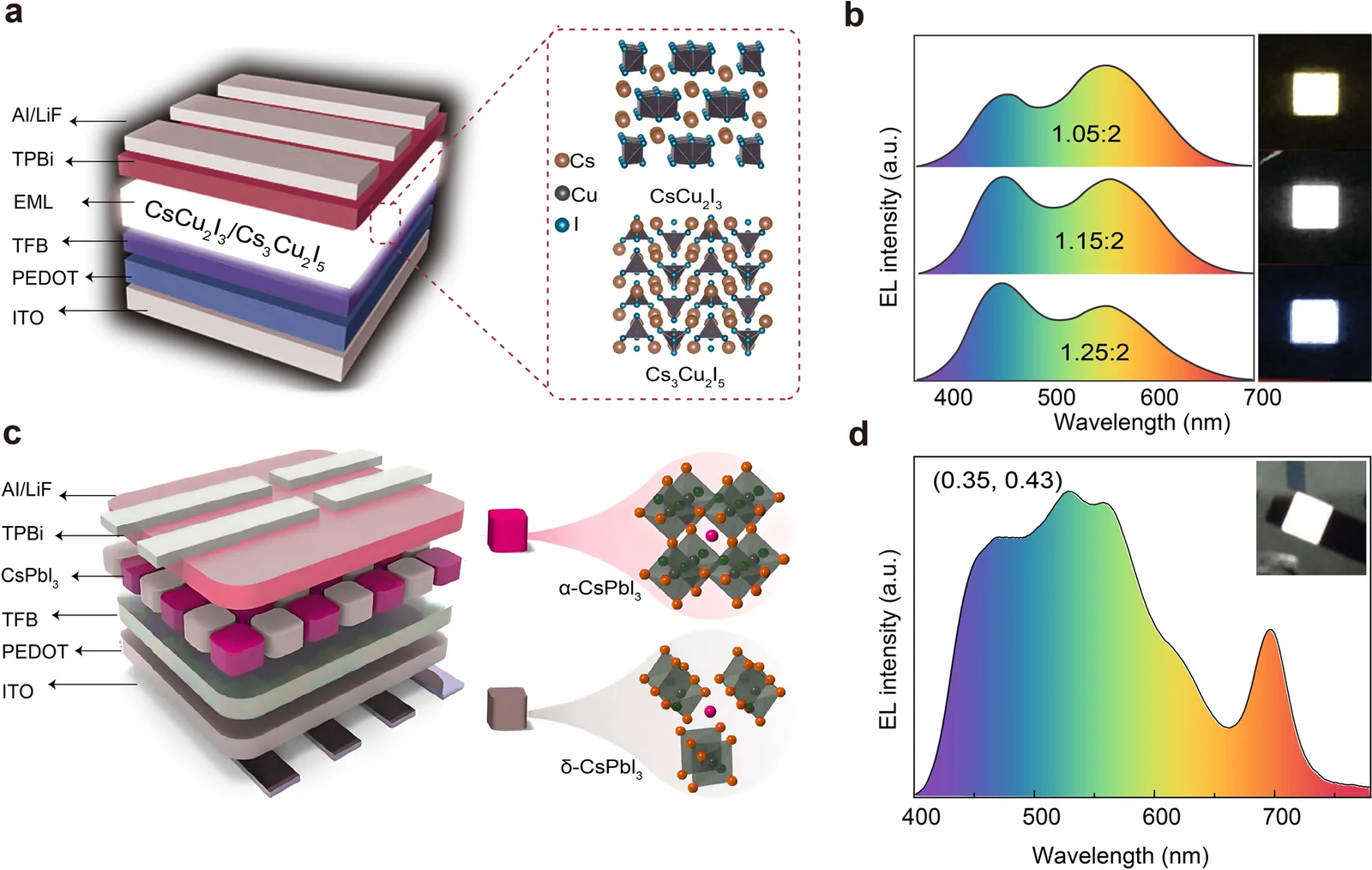
Bulk 3D/3D PPHSs.** a** Devices structure of white PPHS LED, the right side displays the crystal structure of the CsCu_2_I_3_ and Cs_3_Cu_2_I_5_ perovskite [59]. Copyright 2021, Wiley–VCH. **b** EL spectra of the white LED fabricated with different CsI/CuI molar ratios; the insets show EL images of the LEDs [59]. Copyright 2021, Wiley–VCH. **c** Device structure of white PPHS LED, the right side displays the crystal structure of the α-CsPbI_3_ δ-CsPbI_3_ [62]. Copyright 2021, Springer Nature. **d** EL spectra of the white LED fabricated with α-CsPbI_3_/δ-CsPbI_3_ heterostructure; the insets show EL image of the LED [62]. Copyright 2021, Springer Nature

Using two or more perovskite emitters with different emission wavelengths shows promising applications in white light LEDs with stable spectra and high color rendering index (CRI). PPHS is thus an effective candidate for constructing low-energy-loss white electroluminescent devices. However, directly mixing different perovskite materials inevitably leads to ion migration and energy transfer between the perovskites, making it impossible to achieve stable white emission [58]. The uniformly mixed CsCu_2_I_3_/Cs_3_Cu_2_I_5_ copper-based ternary halide bulk 3D/3D PPHS overcomes these limitations. Both CsCu_2_I_3_ and Cs_3_Cu_2_I_5_ belong to the orthorhombic crystal system, and thermodynamic calculations indicate that the two phases can coexist in a single chemical reaction. CsCu_2_I_3_ and Cs_3_Cu_2_I_5_ have bandgaps of 4.0 and 4.09 eV, respectively [59]. Structural distortion leads to broadband emission with energy centers at 2.37 eV and 2.64 eV, respectively, via self-trapped exciton (STE) emission [60, 61]. The independent emission characteristics of the two components in the CsCu_2_I_3_/Cs_3_Cu_2_I_5_ PPHS eliminate the energy transfer and photon self-absorption, making it feasible to achieve stable white emission. Based on this, Ma et al. obtained bulk CsCu_2_I_3_/Cs_3_Cu_2_I_5_ PPHSs with different component ratios through a simple solution method (Fig. 4a) [59]. The CsCu_2_I_3_/Cs_3_Cu_2_I_5_ heterojunction material exhibits excellent white emission performance, with a PLQY up to 50%, and demonstrates outstanding thermal and photostability. By adjusting the molar ratio of CsI/CuI, continuous tuning of cool and warm white light can be achieved, with CIE (Commission Internationale de l’Eclairage) color coordinates varying from (0.21, 0.22) to (0.38, 0.42) (Fig. 4b). White light PeLEDs based on this PPHS material achieve a CRI of 91.6, an EL brightness of 145 cd m^−2^, a peak EQE of 0.15%, and a *T*_50_ of 238.5 min.

Additionally, precisely controlled phase transition can also yield bulk perovskite heterojunctions, enabling the construction of multi-band, white PeLED devices. For example, Chen et al. achieved a controlled phase transition from *α*-CsPbI_3_ to *δ*-CsPbI_3_ by annealing *α*-CsPbI_3_ films at specific temperatures and humidities, resulting in *α*/*δ*-CsPbI_3_ mixed-phase heterojunction films (Fig. 4c) [62]. This heterojunction film combines the excellent charge transport properties of *α*-CsPbI_3_ with the broad white STE emission properties of *δ*-CsPbI_3_. The proportion of *δ*-CsPbI_3_ can increase with rising temperature and can be well stabilized at room temperature through rapid cooling. Therefore, the *α*/*δ* phase ratio can be precisely controlled. LED devices based on this single-layer PPHS film achieve emission characteristics close to natural light, with CIE coordinates at (0.35, 0.43) at 5.2 V (Fig. 4d). The device achieves a maximum brightness of 12,200 cd m^−2^ and a peak EQE of 6.5%.

### 2D/3D PPHSs

3D perovskites possess low exciton binding energy and long charge diffusion distance, making them prime candidates for high-efficiency photovoltaic devices. However, high-efficiency LED devices require effective confinement of electron hole pairs to increase the radiative recombination rate. Additionally, the large number of defect states on the surface of 3D perovskite grains provides abundant non-radiative recombination sites. These characteristics cause the efficiency of 3D PeLEDs to lag behind that of low-dimensional PeLEDs generally [63–65]. Layered 2D perovskites feature wide bandgaps, high exciton binding energies, and high environmental stability. Introducing 2D perovskites onto the surface of 3D perovskites can create an efficient energy funnel, transferring charge carriers into the 3D perovskite interior for recombination, thereby enhancing radiative recombination efficiency. Additionally, the abundant surface ligands of 2D perovskites further enhance the environmental stability and phase stability of 3D perovskites, delaying their decomposition.

#### Vertical Structure

Vertical architecture is the most typical spatial distribution mode for 2D/3D PPHSs. Spin coating a second phase onto pre-deposited perovskite is the most commonly used method for preparing 2D/3D PPHS films. Zhang et al. reported a highly efficient deep red PeLED based on a vertical 2D/3D perovskite heterojunction using a two-step spin-coating process [66]. They first deposited 3D CsPbI_x_Br_3-x_ films through spin coating, then spin-coated p-FPEAI (fluorine-substituted phenyl ethyl ammonium iodide, in ethyl acetate solvent) onto the prepared 3D perovskite film surface. The resulting PPHS film features a top component dominated by layered perovskite with *n* = 2 (*n* denotes the number of inorganic layers). The films treated with p-FPEAI exhibit enhanced PLQY, attributed to the effective passivation of surface defects by the 2D phase and efficient exciton transfer to the 3D phase via energy transfer, thereby enhancing radiative recombination. Transient absorption measurements reveal that the decay time of the 2D phase ground state bleaching signal is significantly longer than the establishment time of the 3D phase bleaching signal after carrier excitation, indicating energy transfer from the *n* = 2 phase to the 3D phase. The optimized PeLED device exhibits a stable EL peak at 674 nm, with a maximum EQE of 22.2%, and significantly extended device operational lifetime.

**Fig. 5:**
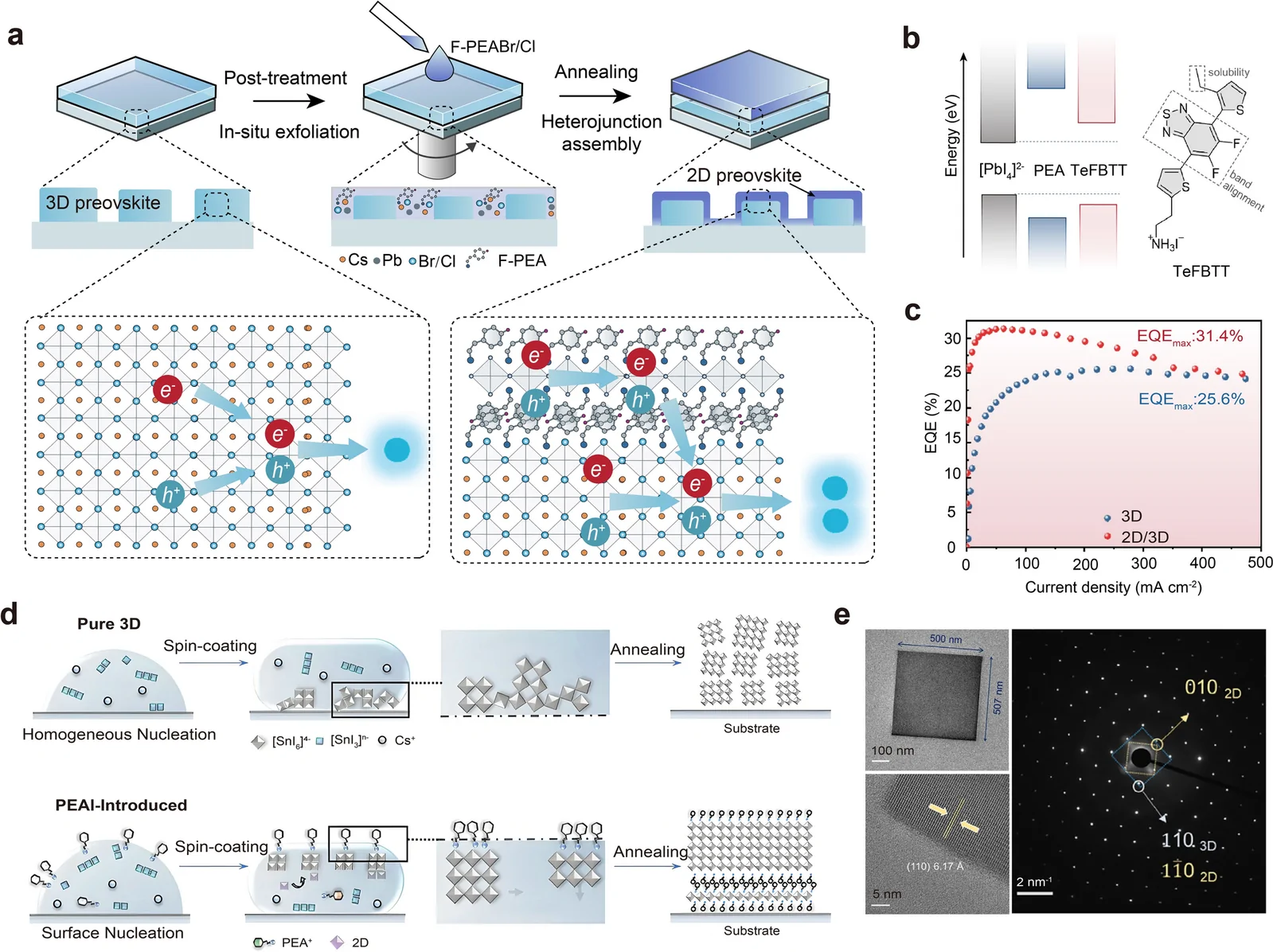
Vertical 2D/3D PPHS.** a ** Schematic showing the fabrication procedure for the F-PEA·CsPbBr_x_Cl_3-x_/CsPbBr_x_Cl_3-x_ 2D/3D PPHS, the bottom showing the carrier transfer and recombination process [67]. Copyright 2024, American Chemical Society.** b** Energy-level alignments between the [PbI_4_]^2−^ inorganic layer and different organic ligands; the right side displays the chemical structure of the TeFBTT ligand [68]. Copyright 2020, Springer Nature.** c** EQE–current density curves of the PeLEDs based on 3D and 2D/3D emission layers. Reproduced with permission [68]. Copyright 2020, Springer Nature.** d** Schematic showing the fabrication procedure for the heteroepitaxial PEA_2_Cs_n−1_Sn_n_I_3n_/CsSnI_3_ 2D/3D PPHS [71]. Copyright 2025, Wiley–VCH.** e** TEM images of the CsSnI_3_ microcube and selected area electron diffraction pattern along [001] of PEA_2_Cs_n−1_Sn_n_I_3n_/CsS_n_I_3_ 2D/3D PPHS [71]. Copyright 2025, Wiley–VCH

The 2D/3D heterojunction, based on a 2D cover layer, has also been proven to significantly reduce the surface trap density, enhance radiative recombination, and suppress halide migration in wide-bandgap 3D blue perovskite. Zhang et al. obtained blue PeLED with an EQE of 12.3%, a full width at half maximum (FWHM) of only 17 nm, and high spectral stability by spin-coating p-FPEABr/Cl onto the surface of 3D CsPbBr_3-x_Cl_x_ perovskite (Fig. 5a) [67]. The improvement in device performance stems from the in situ growth of 2D perovskite, which partially dissolves and removes surface components, reduces halide vacancies and uncoordinated Pb^2+^ defects, and enables rapid energy transfer from the 2D to 3D phase, thereby suppressing non-radiative recombination. Additionally, they demonstrated that the 2D coating inhibits halide migration in mixed halide recombination centers.

Band alignment in 2D/3D PPHS is a key factor influencing carrier injection and recombination in LED devices. The molecular structure of organic cations in 2D perovskites is a critical factor affecting the electronic energy levels and band alignment in heterojunctions. Baek et al. demonstrated that while 2D perovskites with the commonly used phenethylamine (PEA) cation form a type-I band alignment with 3D components, enabling effective carrier confinement, they exhibit high-energy barriers for carrier injection, which is disadvantageous for LEDs [68]. On the other hand, some highly conjugated long-chain ligands, such as trithiophenylethylammonium (3T), form type-II band alignment with the inorganic layer of perovskite, which is also unfavorable for the realization of efficient LEDs due to low carrier confinement capability. Therefore, they designed a narrow-bandgap organic cation TeFBTT, where the fluorobenzothiadiazole (FBT) core confers a deep highest occupied and lowest unoccupied molecular orbitals (HOMO and LUMO) of TeFBTT (Fig. 5b). The TeFBTT shows HOMO and LUMO energy levels closely aligned with the valence band and conduction band of the [PbI_4_]^2−^ inorganic layer, while ensuring type-I band alignment. The perovskite film with TeFBTT 2D top layer exhibited efficient defect passivation, enhanced energy transfer efficiency, and improved carrier injection. The final PeLEDs achieved a peak EQE of 31.4% at 798 nm and a maximum radiant brightness of 929 W sr^−1^ m^−2^, with performance comparable to that of commercial III-V semiconductor devices (Fig. 5c).

Vertical 2D/3D perovskite heterostructures can also be formed through self-assembly. Zhu demonstrated that 3D inorganic perovskites, such as CsPbBr_3_ and Cs_2_AgBiBr_6_, can be post-grown and oriented on the surface of 2D perovskites containing organic amine cations, forming epitaxially grown 2D/3D perovskite heterostructures [69]. The organic amines can be either aliphatic or aromatic, such as PEA, butylamine (BA), phenylmethylamine (PMA), and 1-naphthylmethylamine (NMA). Their research indicates that the key to achieving the epitaxial self-assembly of perovskite heterostructures lies in the replacement of the long-chain ligands (such as oleic acid and oleylamine) covering the CsPbBr_3_ cubes with organic amine cations (such as PEA) in the reaction solution. Taking PEA as an example, the heterointerface between the ligand-assisted self-assembled 2D/3D PPHSs exhibits a gap of approximately 1.7–1.8 nm, similar to the (001) plane interlayer spacing of the PEA_2_PbBr_4_, indicating that the heterointerface consists of two layers of PEA molecules. Transmission electron microscopy (TEM) and selected area electron diffraction also confirm the epitaxial arrangement of CsPbBr_3_ cubes on PEA_2_PbBr_4_ plates. In contrast, the 2D/3D heterostructure with long-chain OA/OLA ligands that were not effectively substituted exhibited a relatively large interfacial gap of 2–5 nm, along with the absence of crystallographic coordination. They also demonstrated that the epitaxial heterostructure exhibits efficient charge and energy transfer, whereas the randomly assembled heterostructure has lower charge transfer efficiency due to the obstruction caused by disordered ligand layers.

Self-assembled 2D/3D vertical HPPSs were also realized in tin-based perovskites. Min et al. obtained films with coexisting top-layer three-dimensional FA_0.9_Cs_0.1_SnI_3_ perovskite and bottom-layer horizontally oriented 2D (*n* = 1 and 2) perovskite by adding tryptophan (Trp) and SnF_2_ additives during a one-step spin-coating process [70]. In this structure, the crystal lattices of the 2D layers and 3D crystals are aligned vertically, indicating an epitaxial growth. The key to forming this vertical epitaxial structure lies in the ability of SnF_2_ and Trp additives to induce the sequential crystallization of 2D and 3D phases, with the 2D perovskite self-assembling in situ on the 3D phase during the annealing stage. The resulting films exhibit highly ordered structures and distinct interfaces, significantly reducing defects and non-radiative recombination. Additionally, due to the low valence band offset and high conduction band offset between the bottom 2D perovskite and the 3D composite center, it serves as an ideal hole injection and electron blocking layer. Therefore, they directly used the 2D perovskite layer as the carrier transport layer, achieving a peak EQE of 11.6% for the tin-based PeLED. Similarly, Ye et al. achieved heteroepitaxial growth of 3D CsSnI_3_ on 2D perovskite substrates by introducing PEA^+^ into the CsSnI_3_ precursor solution, yielding highly oriented micrometer-scale single-crystal cubes (Fig. 5d, e). This structure effectively suppressed tin oxidation and minimized defect density. Ultimately, the fabricated tin-based infrared LED achieved a radiant luminance of 152 W sr^−1^ m^−2^ and a peak EQE of 8.11% [71].

#### Lateral Structure

2D perovskite layers perpendicular to the substrate can be embedded within oriented 3D perovskite grains, forming a 2D/3D lateral stacked heterostructure. For example, Wang et al. observed the vertical alignment of 2D phase crystals between 3D perovskite grains by introducing BA cations into FA_0.83_Cs_0.17_PbI_3y_Br_3−3y_ perovskite [72]. The reason for the formation of this vertically oriented 2D perovskite layer is that BA cations are expelled from the 3D perovskite domains during crystallization and subsequently grow along the (010) plane of 3D perovskite perpendicular to the substrate. Since the in-plane direction of the 2D perovskite is highly aligned with the (010) plane of the 3D perovskite lattice, the vertical orientation of the 2D perovskite is thermodynamically and kinetically favorable. Similarly, by spin coating a solution of bulk-phase perovskite precursors onto pre-deposited vertically oriented 2D perovskite seeds, oriented 3D perovskite growth can be induced, resulting in a 2D/3D vertically stacked heterostructure. This vertically oriented 2D perovskite effectively passivates the 3D perovskite grain boundaries. Additionally, the 2D phases promote charge extraction and transport [73]. Solar cells based on this heterostructure have achieved high power conversion efficiency (PCE) and fill factor. However, these heterostructures have rarely been applied to LED devices, possibly due to the lower thickness of the LED active layer, which makes vertical growth of 2D perovskite challenging.

#### Bulk Structure

During the synthesis of 3D APbX_3_ perovskites, in the case of PbX_2_ enrichment, APb_2_X_5_/APbX_3_ 2D/3D PPHSs can also be yielded. Among these, the 2D/3D heterojunction formed by CsPb_2_Br_5_ and CsPbBr_3_ has been widely reported. CsPb_2_Br_5_ is a 2D all-inorganic lead halide perovskite with edge-sharing octahedral, exhibiting excellent chemical stability [74]. As a wide-bandgap indirect bandgap semiconductor material with a bandgap exceeding 3.0 eV, CsPb_2_Br_5_ lacks intrinsic fluorescence. However, CsPb_2_Br_5_ can form type-I heterojunctions combined with small-bandgap perovskites such as CsPbBr_3_. In this PPHS, the CsPb_2_Br_5_ capping layer can reduce trap density and exciton diffusion length in CsPbBr_3_, construct an energy transfer funnel, and suppress non-radiative recombination induced by surface trap sites [75, 76]. Additionally, CsPb_2_Br_5_ can serve as an ideal protective layer to enhance perovskite stability. Currently, researchers have demonstrated that synthesis processes, such as hot injection [77], ligand-assisted precipitation [78], and mechanical grinding [79], can promote the conversion of CsPbBr_3_ to CsPb_2_Br_5_. For example, Liu et al. triggered structural reorganization of the CsPbBr_3_ surface via water molecules, converting it into the more stable CsPb_2_Br_5_ [31]. The resulting CsPb_2_Br_5_/CsPbBr_3_ heterojunction exhibits excellent environmental stability, retaining high optical activity even after two months of immersion in water, and demonstrates the potential application in high-efficiency, high-CRI white LEDs. Wang et al. introduced Na^+^ into the precursor solution to compete with Pb^2+^, inhibiting the rapid formation of 3D perovskite phases, allowing Pb^2^⁺ to be released during annealing and to form CsPb_2_Br_5_ [80]. Based on the passivation effect of CsPb_2_Br_5_ on CsPbBr_3_ and the formation of a type-I band structure, a blue PeLED with a peak EQE of 12.86% was demonstrated.

### 0D/3D PPHSs

**Fig. 6:**
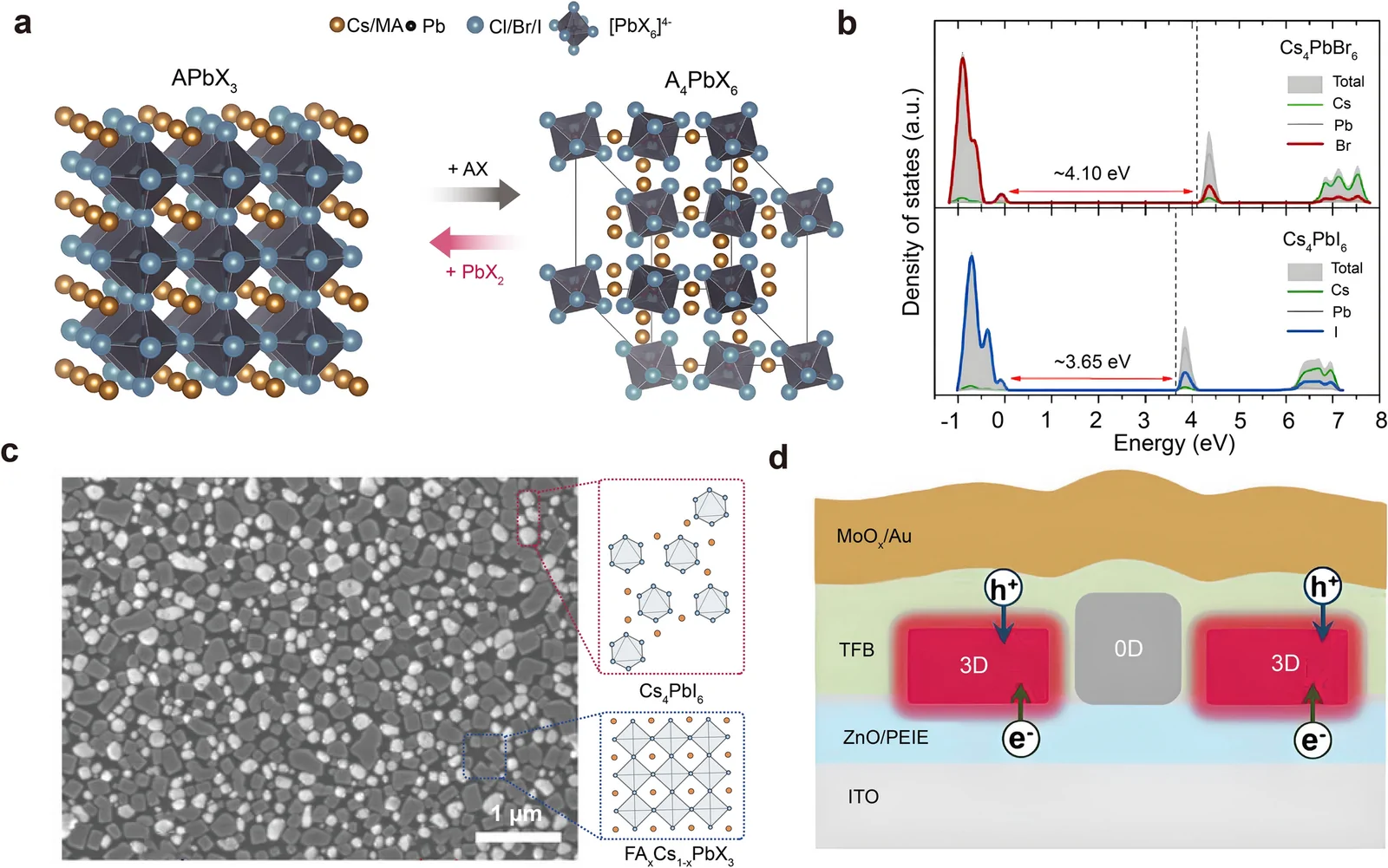
Vertical 0D/3D PPHSs.** a** Crystal structure of 3D cubic APbX_3_ and 0D hexagonal A_4_PbX_6_ perovskites. **b** Density of states and forbidden bandwidth of Cs_4_PbX_6_ perovskites [81]. Copyright 2023, American Chemical Society. **c** SEM image and schematic diagram of the Cs_4_PbI_6_/FA_x_Cs_1-x_PbI_3_ 0D/3D PPHS [82]. Copyright 2024, Wiley–VCH. **d** Schematic showing the device structure and carrier injection in the PeLED based on the 0D/3D PPHS [82]. Copyright 2024, Wiley–VCH

As mentioned earlier, 0D perovskites can be categorized into two types, based on crystal structure and morphology. A_4_PbX_6_ perovskite possesses spatially disconnected [PbX_6_]^4–^ octahedra, which refers to 0D perovskite in terms of its crystal structure (Fig. 6a). Morphological 0D perovskite nanocrystals can achieve bandgap tuning through the quantum confinement effect, and feature increased exciton binding energy and recombination rate. However, the high specific surface area of 0D nanocrystals significantly increases the probability of charge carriers being trapped at surface defects, thereby reducing luminescence efficiency and material stability. Combining 2D perovskites with 3D counterparts, such as embedding perovskite QDs into a 3D perovskite matrix—particularly under conditions of crystal extension alignment—has been demonstrated to enhance carrier transport efficiency, accelerate radiative recombination rates, and improve material stability.

#### Lateral Structure

A_4_PbX_6_ perovskite emerges as a by-product of APbX_3_ perovskite and can spontaneously form A_4_PbX_6_/APbX_3_ 0D/3D perovskite heterostructures by adjusting the ratio of AX and PbX_2_ in the precursor. Although it has been reported that A_4_PbX_6_ exhibits a high PLQY, the origin of A_4_PbX_6_’s fluorescence remains a topic of debate. Furthermore, the complete decoupling of the [PbX_6_]^4–^ octahedra in all dimensions within the A_4_PbX_6_ perovskite results in increased bandgaps (Cs_4_PbCl_6_ ~ 4.37 eV, Cs_4_PbBr_6_ ~ 4.10 eV, and Cs_4_PbI_6_ ~ 3.65 eV) (Fig. 6b) [36, 81], making such materials unsuitable for constructing electroluminescent devices. However, an increasing number of studies have shown that A_4_PbX_6_/APbX_3_ 0D/3D perovskite heterostructures play a significant role in passivating 3D recombination centers and enhancing recombination rates and have been utilized to construct efficient PeLED devices. A_4_PbX_6_/APbX_3_ 0D/3D perovskite heterostructures have been proven to form horizontal arrangements due to their sequential crystallization characteristics. Ke et al. introduced excess CsI into the perovskite precursor solution and obtained Cs_4_PbI_6_/FA_x_Cs_1-x_PbI_3_ perovskite heterostructures via a one-step spin-coating process, where the protruding, irregularly shaped Cs_4_PbI_6_ grains are laterally dispersed alongside rectangular FA_x_Cs_1-x_PbI_3_ grains (Fig. 6c, d) [82]. They found that excess CsI tends to form 0D Cs_4_PbI_6_ in the initial stage, followed by the formation of FA-rich FA_x_Cs_1-x_PbI_3_ perovskite. This process yields 3D perovskite with high crystallinity and fewer grain boundaries, thereby reducing defects while suppressing the formation of non-perovskite *δ*-CsPbI_3_. Additionally, Cs_4_PbI_6_ has a refractive index significantly lower than that of FA_x_Cs_1-x_PbI_3_. The laterally dispersed 0D perovskite thus acts as a grating structure, significantly enhancing light scattering within the device and facilitating light extraction. The resulting PeLED achieves deep red emission at 705 nm, with a peak EQE of 21% and a high EQE of 20.5% even at a current density of 200 mA cm^−2^.

#### Bulk Structure

**Fig. 7:**
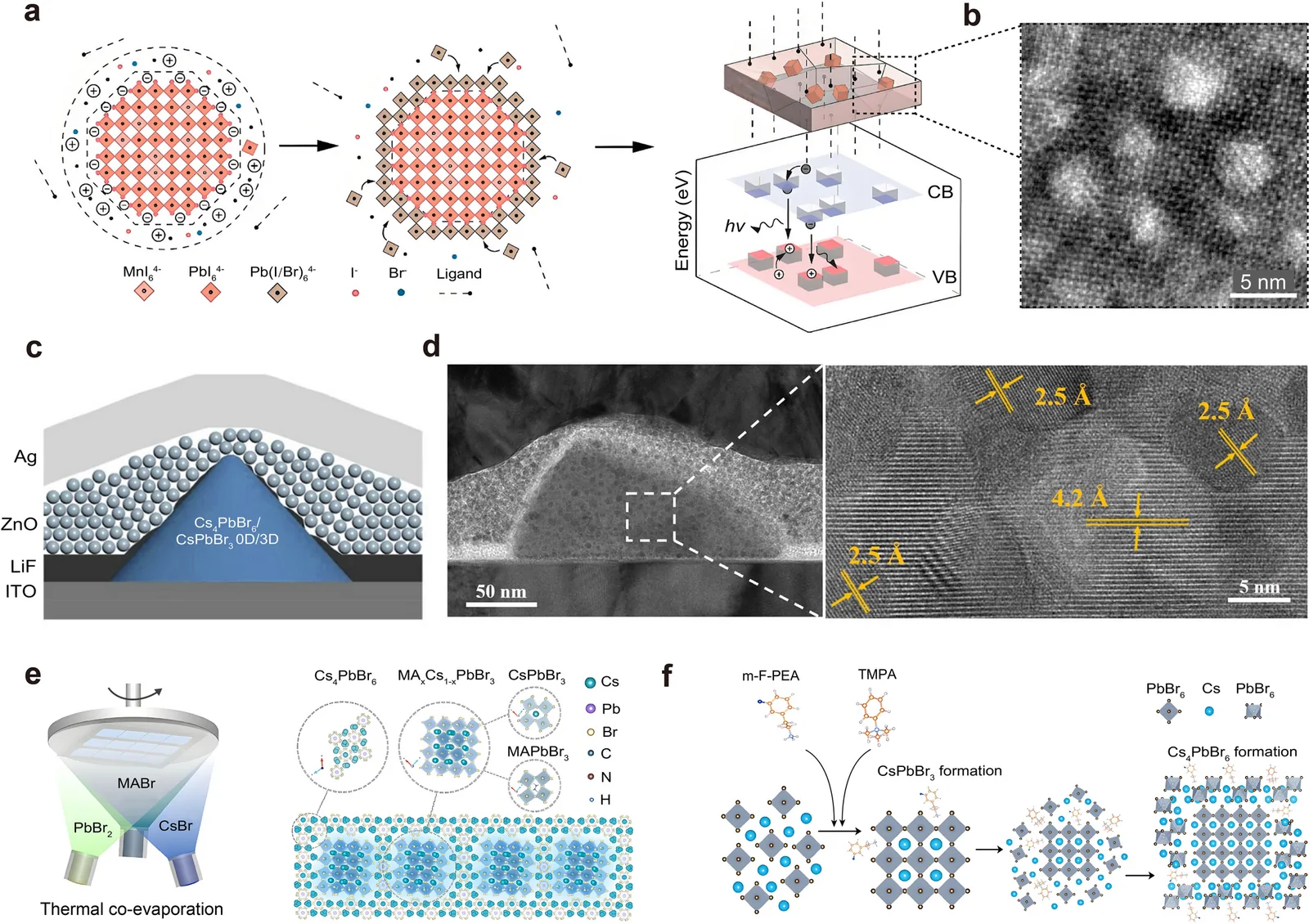
Bulk 0D/3D PPHSs.** a** Schematic showing the CsPb(Br_x_I_1-x_)_3_ perovskite matrix crystallization along the Mn-doped CsPbI_3_ QDs [85]. Copyright 2021, American Chemical Society. **b** TEM image of CsPbI_3_ QDs in CsPb(Br_x_I_1-x_)_3_ matrix [85]. Copyright 2021, American Chemical Society. **c, d** Structure diagram and cross-sectional TEM images of the Cs_4_PbBr_6_/CsPbBr_3_ 0D/3D PeLED device [86]. Copyright 2024, American Chemical Society. **e** Schematic showing the Cs_4_PbBr_6_/MA_x_Cs_1-x_PbBr_3_ 0D/3D PPHS film fabricated through thermal co-evaporation [87]. Copyright 2025, Wiley–VCH. **f** Schematic showing the crystallization kinetics process of the Cs_4_PbBr_6_/CsPbBr_3_ 0D/3D PPHS film [88]. Copyright 2025, Wiley–VCH

Growing heteroepitaxial semiconductors on the surface of 0D nanocrystals is a feasible approach to enhance emission efficiency and device stability, as validated in II-VI and III-V core/shell QDs [83, 84]. Compared to traditional II-VI and III-V nanocrystal heterojunctions, constructing heterojunctions based on 0D perovskites requires overcoming three challenges: 1) The structural instability of perovskite materials due to their ionic crystal nature, particularly ion migration during the solution process. 2) The abundance of surface ligands on 0D perovskite nanocrystals makes it challenging to achieve high-quality epitaxial growth with the second phase. 3) Lattice mismatch issues between 0D perovskite nanocrystals and the epitaxial phase. Based on this, Liu et al. achieved stable, short-chain ionic ligand surface-capped CsPbI_3_ 0D QDs in polar solvents through surface ligand treatment [85]. Specifically, they obtained surface electrostatically stabilized CsPbI_3_ QDs through sequential isopropyl ammonium iodide and KI treatment. Additionally, they introduced lattice contraction of the QDs by doping Mn^2+^, which not only enhanced the stability of the QDs but also enabled lattice matching with the wide-bandgap CsPb(Br_x_I_1-x_)_3_ perovskite. By adding the QD solution during the spin-coating process of the CsPb(Br_x_I_1-x_)_3_ precursor solution, they obtained a 0D/3D heteroepitaxial structure with CsPbI_3_ QDs embedded in the CsPb(Br_x_I_1-x_)_3_ matrix, with high-quality epitaxial alignment (Fig. 7a, b). The PL spectrum of this 0D/3D PPHS only exhibited pure-red emission from the 0D quantum dots and attributed to efficient charge transfer from the wide-bandgap matrix to the 0D QD emitters. The 0D/3D bulk PPHS also exhibits high stability. Under a high excitation energy density of 600 W cm^−2^, the PLQY of the QD-in-matrix thin film remains at 40%, while the single-phase QD thin film undergoes severe optical deactivation. The obtained red PeLED exhibits an EQE of 18% and a maximum luminance of 4700 cd m^−2^. Even under high injection of 100 mA cm^−2^, the devices’ EQE remains at 13%, attributed to the matrix reducing the non-radiative Auger recombination. The reduced Auger recombination can be attributed to the wide-bandgap matrix as a carrier diverter. Injected carriers are first captured by the wide-bandgap phase and then transported quantitatively to the narrow-bandgap emission center via energy transfer or charge injection, significantly reducing the transient carrier density under high injection. More importantly, the obtained PeLED exhibits excellent stability, with a *T*_50_ of 2400 h at an initial luminance of 100 cd m^−2^, which is two orders of magnitude higher than the best value reported for previous pure-red PeLEDs. This QD-in-3D-matrix heterostructure also demonstrates feasibility in constructing blue devices. To screen for a 3D matrix compatible with blue CsPbBr_3_ QDs while arranging a type-I band structure, they employed Sr^2+^-alloyed CsPb_1−x_Sr_x_Br_3_ perovskite as the matrix [32]. Since the ionic radii of Sr^2+^ and Pb^2+^ are similar (1.18 Å vs. 1.19 Å), this avoids lattice mismatch between the QDs and the 3D matrix. Additionally, Sr^2^⁺ doping increases the bandgap of the perovskite matrix, with a bandgap width of 2.6 eV, higher than that of CsPbBr_3_ QDs (2.55 eV). To address the inherent hygroscopic nature of Sr, they also introduced a bis(4-fluorophenyl)phenyl phosphine oxide (DFPPO) additive to passivate the CsPb_1−x_Sr_x_Br_3_ surface, significantly enhancing the stability of the 0D/3D PPHS thin film in air. The blue PeLEDs based on CsPbBr_3_/CsPb_1−x_Sr_x_Br_3_ 0D/3D heterostructure exhibit high efficiency and spectral stability. The device achieved a peak EQE of 13.8% and a peak luminance exceeding 6000 cd m^−2^ at an emission wavelength of 495 nm, with no significant spectral shift observed even under an external bias voltage as high as 12 V.

A_4_PbX_6_/APbX_3_ 0D/3D bulk PPHSs have also been widely applied in high-performance PeLEDs. Additionally, compared to lateral and vertical 0D/3D PPHSs, A_4_PbX_6_/APbX_3_ 0D/3D bulk PPHSs are easier to obtain. A representative example is the Cs_4_PbBr_6_/CsPbBr_3_ 0D/3D bulk heterojunction. Gong et al. developed a carrier-confined bulk heterojunction centered on CsPbBr_3_ 3D crystals with a size of tens of nanometers, surrounded by a wide-bandgap 0D Cs_4_PbBr_6_, by increasing the concentration of CsBr in the precursor (CsBr/PbBr_2_ = 1.4) (Fig. 7c, d) [86]. They demonstrated that the Cs_4_PbBr_6_/CsPbBr_3_ 0D/3D heterostructure forms during thin-film annealing, as excess CsBr causes the CsBr-rich regions around the CsPbBr_3_ and promotes the generation of Cs_4_PbBr_6_. This PPHS avoids surface defect state capture while increasing the volume of the radiative recombination center. The 3D CsPbBr_3_ emitter achieves low Auger recombination due to its larger crystal size, while the wide-bandgap Cs_4_PbBr_6_ promotes carrier confinement. The Cs_4_PbBr_6_/CsPbBr_3_ 0D/3D bulk heterostructure thus demonstrates significant potential for enhancing radiative recombination. Based on a simple all-inorganic structure, the obtained PeLED exhibits a peak EQE of 17.0% and a maximum luminance exceeding 100,000 cd m^−2^. Additionally, this device maintains 92% and 74% of the maximum EQE values at brightness levels of 100,000 cd m^−2^ and 200,000 cd m^−2^, respectively. In addition to the solution method, A_4_PbX_6_/APbX_3_ 0D/3D bulk PPHSs can also be obtained through vapor deposition processes (Fig. 7e) [29, 87]. By increasing the CsBr/PbBr_2_ molar ratio to 1.56 during thermal evaporation, Du et al. obtained large-area Cs_4_PbBr_6_/CsPbBr_3_ PPHS films [29]. The PL peak of the film is located at 508 nm, significantly lower than that of bulk CsPbBr_3_ (> 520 nm), indicating that electrons and holes are strongly spatially confined in the CsPbBr_3_ phase, which serves as the emissive center, surrounded by Cs_4_PbBr_6_. They demonstrated that the introduction of Cs_4_PbBr_6_ not only reduces the trap-assisted recombination rate but also significantly enhances the bimolecular recombination rate, thereby improving PeLEDs’ performance. Based on a thermally evaporated Cs_4_PbBr_6_/CsPbBr_3_ heterojunction thin film, the obtained PeLEDs show a peak EQE of 8.0%, and an EQE of 7.1% was realized in a large-area device of 40.2 cm^2^, demonstrating excellent scalability.

In the aforementioned examples, the Cs_4_PbBr_6_/CsPbBr_3_ 0D/3D perovskite heterostructures are obtained through thermodynamic control, i.e., by introducing an excess of CsBr to enhance the thermodynamic stability of Cs_4_PbBr_6_. However, due to the low charge transport capability of Cs_4_PbBr_6_, the relative content of CsPbBr_3_ and Cs_4_PbBr_6_ must be carefully adjusted to achieve effective charge injection. Therefore, to obtain Cs_4_PbBr_6_/CsPbBr_3_ heterostructure with both excellent optical and electrical properties, researchers also explored precisely controlling the kinetic processes of different components during the liquid-phase process. Due to competition between 3D CsPbBr_3_ and 0D Cs_4_PbBr_6_ during in situ growth, precise control of the crystallization sequence of different perovskite phases can yield the Cs_4_PbBr_6_/CsPbBr_3_ heterostructure with low 0D/3D ratios at low CsBr addition levels. Xia et al. proposed a co-addition method to regulate the crystallization of perovskite [88]. They introduced two additives, m-F-PEA and TMPA (trimethylphenylammonium bromide), where the former reduces surface energy by adsorbing on the perovskite surface, while the latter increases surface energy (Fig. 7f). The high affinity of m-F-PEA with perovskite reduces nucleation barriers and promotes the crystallization of CsPbBr_3_, which serves as the core for the Cs_4_PbBr_6_ phase. TMPA, on the other hand, distorts the perovskite crystal structure and delays the crystallization process, inducing the in situ formation of Cs_4_PbBr_6_ on the already formed CsPbBr_3_ surface. Based on this co-addition technique, the non-radiative recombination rate of the obtained heterostructure film was reduced by 14 times, with a PLQY approaching unity. Additionally, the hole and electron mobility of the obtained films were significantly improved, attributed to the optimized Cs_4_PbBr_6_/CsPbBr_3_ ratio and TMPA surface doping, which transformed the perovskite from n to p-type, facilitating more balanced electron/hole injection. The PeLED employing the co-additive strategy exhibits a peak EQE of 28.2% and a maximum luminance exceeding 150,000 cd m^−2^, with EQE exceeding 20% across a wide brightness range from 10 to 20,000 cd m^−2^. More importantly, the device exhibits an estimated *T*_50_ exceeding 4200 h at 100 cd m^−2^. This outstanding stability stems from balanced electron/hole injection, reduced Joule heating, and enhanced film stability due to Cs_4_PbBr_6_.

### 0D/2D PPHSs

0D/2D PPHSs can be formed via a two-step spin-coating method or self-assembly. In this case, the 0D perovskite serves as the radiative recombination center, while the 2D phase functions as a protective layer and energy transfer medium. The 0D/2D PPHSs enable efficient energy cascade transfer, significantly enhancing radiative recombination efficiency while reducing non-radiative recombination. Furthermore, utilizing 2D perovskites has been demonstrated to stabilize metastable 0D QDs. Through rational ligand design, in situ construction of 0D/2D heteroepitaxial structures is achievable. This epitaxial interface suppresses inactive phase transitions by inducing lattice distortion while simultaneously enhancing the film’s charge transport capability and thermodynamic stability.

#### Vertical Structure

Li et al. demonstrated the preparation of 0D/2D vertical PPHS via a two-step spin-coating process, where FAPbI_3_ QDs in a non-polar solvent were spin-coated onto a pre-deposited quasi-2D perovskite film [89]. The narrow-bandgap FAPbI_3_ QDs serve as the radiative recombination center, while the wide-bandgap quasi-2D phase acts as the protective layer. The obtained 0D/2D PPHS exhibits excellent stability, reduced Auger recombination rates, and low electron–phonon coupling strength. As a result, they achieved the near-infrared PeLEDs with a peak EQE of 21.82%. 0D/2D PPHSs can also be formed through a self-assembly process. It is worth noting that due to the reduction in size of 0D perovskite, 0D/2D PPHS exhibits easier random orientation and distribution in space. Thus, the 2D perovskite phase in self-assembly 0D/2D vertical PPHS typically has larger lateral dimensions to facilitate layering of 0D/2D PPHSs in the vertical direction. Chin et al. demonstrated that introducing n-octylamine during the synthesis of FAPbBr_3_ nanocrystals using the ligand-assisted reprecipitation (LARP) method can promote the formation of micron-scale (OA)_2_(FA)_n-1_Pb_n_Br_3n+1_ 2D layered perovskites [90]. In the obtained films, micron-sized 2D phases were observed deposited on top of 0D FAPbBr_3_ nanocrystals. They proposed that this self-assembly is induced by surface energy and wettability, where 2D phases may aggregate at the liquid–gas interface due to their larger ligand-to-surface ratio. This self-assembled hierarchical nanostructure enables an efficient energy cascade mechanism, where the wide-bandgap 2D phase (~ 2.82 eV) acts as an energy donor, rapidly transferring excitons to 0D nanocrystals (~ 2.34 eV), significantly enhancing radiative recombination efficiency. They deliver a PeLED with a peak EQE of 13.4% and demonstrate compatibility of the PPHS films with high-efficiency, large-area, and flexible devices.

#### Bulk Structure

**Fig. 8:**
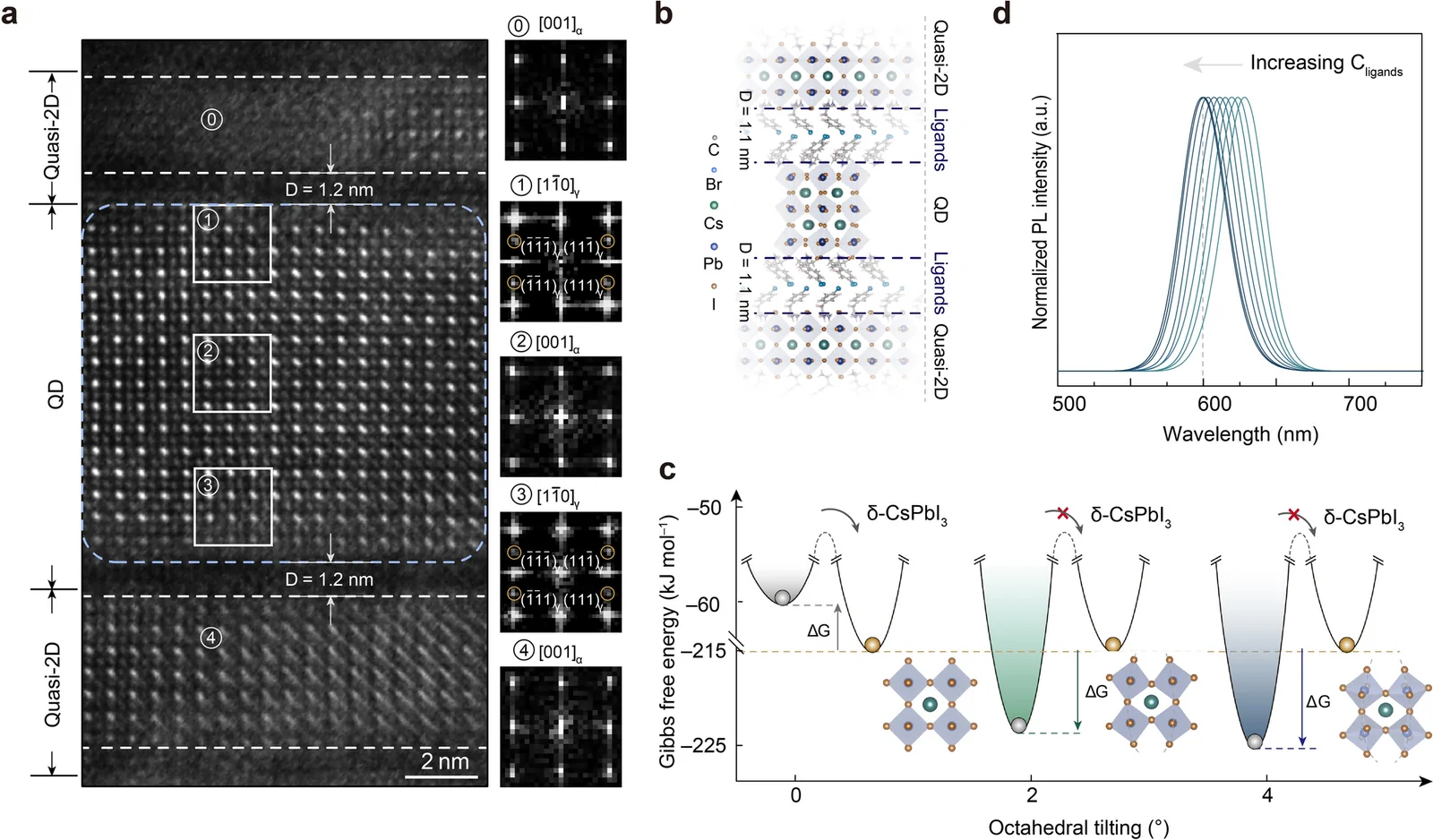
Bulk 0D/2D PPHSs.** a** Atomic-scale TEM image of epitaxial growth CsPbI_3_ QDs/Quasi-2D perovskite 0D/2D PPHS, the right side displays the FFT (fast Fourier transform algorithm) of different regions [91]. Copyright 2025, Springer Nature. **b** Schematic shows the epitaxy structure and the interface spacing of the 0D/2D PPHS [91]. Copyright 2025, Springer Nature. **c** Energy gap of the phase transform between the tilted-CsPbI_3_ and δ-CsPbI_3_; insets show lattice structures of different tilted-CsPbI_3_ [91]. Copyright 2025, Springer Nature. **d** PL spectra evolution of 0D/2D PPHS films as ligand concentration increased from 0.02 M to 0.28 M [91]. Copyright 2025, Springer Nature

**Fig. 9:**
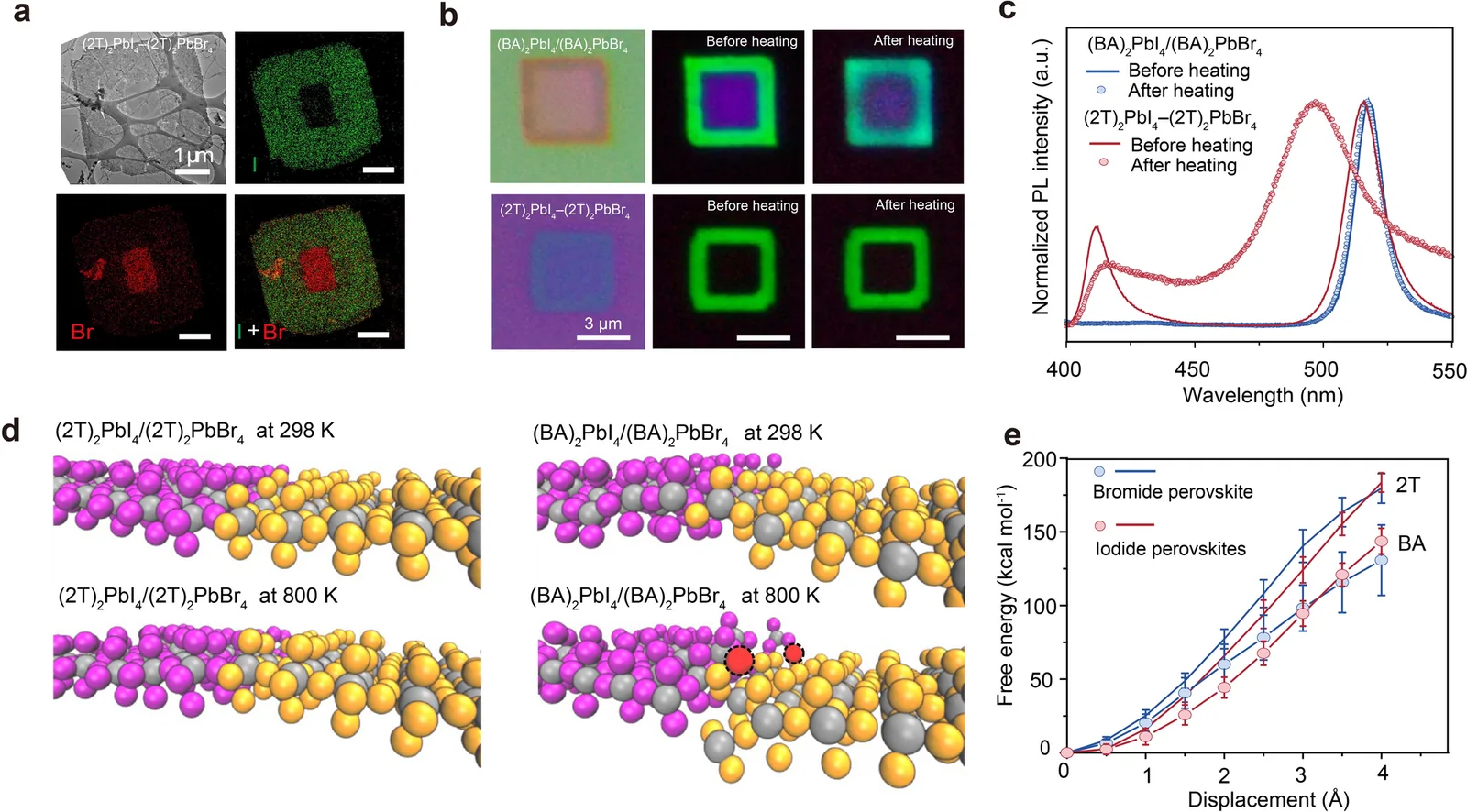
Vertical 2D/2D PPHSs. **a** TEM image and EDS elemental mappings of (2 T)_2_PbI_4_/(2 T)_2_PbBr_4_ PPHS [93]. Copyright 2020, Springer Nature. **b,c** Optical, PL images (b) and PL spectra (c) of (2 T)_2_PbI_4_/(2 T)_2_PbBr_4_ and (BA)_2_PbI_4_/(BA)_2_PbBr_4_ PPHS before and after heating [93]. Copyright 2020, Springer Nature. **d** Molecular dynamics simulations of (2 T)_2_PbI_4_/(2 T)_2_PbBr_4_ and (BA)_2_PbI_4_/(BA)_2_PbBr_4_ PPHSs at different temperatures, the diffusion of iodine atoms across the interface and into the bromine domain is indicated in dashed circles [93]. Copyright 2020, Springer Nature. **e** Halide atom migration free energy in different 2D/2D PPHS [93]. Copyright 2020, Springer Nature

Ultra-small 0D perovskite QDs are the most promising candidates for achieving efficient and stable pure-red and deep blue PeLEDs. However, ultra-small QDs are difficult to maintain their solution-phase properties when assembled into solid conductive films. This is because the abundant insulating ligands on the QDs’ surface hinder charge transport, necessitating their removal during assembly into semiconductor solids. However, the ligand desorption process often leads to QD agglomeration and an increase in surface defects. More seriously, due to reduced surface energy, metastable perovskites such as CsPbI_3_ may transform into photoinactive *δ* phases. Replacing long-chain organic ligands with 2D perovskites and constructing 0D/2D heteroepitaxial structures is a viable strategy for stabilizing 0D perovskite QDs. Wei et al. in situ synthesize QDs/2D perovskite PPHSs on the substrate, through rational ligand selection [91]. They introduced two ligands, Br-DMA^+^ (*α*,*α*-dimethyl-4-bromide-benzyl-ammonium) and Br-PEA^+^, during the spin-coating process. Among them, Br-DMA^+^, which has a large head steric hindrance effect, inhibits the long-range ordered arrangement of octahedrons and promotes the formation of CsPbI_3_ QDs, while Br-PEA^+^, which has a small head steric hindrance, promotes the formation of layered 2D perovskite. The halogen elements at the tails of the two ligands enhance intermolecular dipole–dipole interactions, enabling quasi-2D perovskites epitaxial alignment on the surface of CsPbI_3_ QDs (Fig. 8a, b). They also found that CsPbI_3_ QDs at the heterointerface exhibit significant octahedral tilting, which increases the Gibbs free energy difference between the black photoactive phase and the photoinactive δ phase, thereby enhancing the thermodynamic stability (Fig. 8c). Based on this, they delivered high-quality, stable 0D/2D heterostructure films with tunable emission covering the entire red spectral region (from 600 to 710 nm) (Fig. 9d) and demonstrated a pure-red PeLED with a high peak EQE of 24.6% (EL peak at 630 nm).

### 2D/2D PPHSs

Lateral architecture is the most typical spatial distribution mode for 2D/2D PPHSs, which can be obtained through liquid-phase epitaxial growth. As the nucleation and growth of halide perovskite occur along the edges of the prior 2D crystals, concentric square or rectangular 2D/2D horizontal PPHSs can subsequently form on the single substrate [92]. By controlling solution concentration, growth temperature, and time, the lateral size and vertical thickness of the 2D/2D PPHSs can be controlled. This lateral heterostructure demonstrates potential application value in constructing rectifying thin-film transistors. Similar to vertically structured PPHS, due to the inherently high ionic mobility of halide perovskites, the prepared 2D/2D PPHSs result in spontaneous ionic diffusion and increased junction width, making it challenging to achieve high interface sharpness. Shi et al. reported a strategy to significantly suppress planar ion diffusion in two-dimensional halide perovskites by introducing large-volume rigid *π*-conjugated organic ligands. They demonstrated horizontal 2D/2D PPHSs with near-atomic-level sharp interfaces (Fig. 9a) [93]. Specifically, they utilized a conjugated ligand based on biphenylthioethylammonium (2 T). Take (2 T)_2_PbI_4_/(2 T)_2_PbBr_4_ PPHS as an example, after heating at 100 °C for 1 h, the PL spectrum of the heterojunction showed no significant shift. In contrast, the control heterostructure (BA)_2_PbI_4_/(BA)_2_PbBr_4_ exhibits a blurred two-phase interface after heating, and the PL emission spectrum undergoes significant changes (Fig. 9b, c). The sharp heterojunction interface and high stability stem from the larger and more rigid conjugated organic ligands, which can more effectively stabilize the interface and inorganic framework, suppressing halide interdiffusion at the interface. Smaller organic ligands, however, result in a softer inorganic lattice and promote halide interdiffusion (Fig. 9d, e). This solution-phase epitaxial growth strategy is equally applicable to synthesizing various lateral heterostructures with different halide, metal cation, and organic ligand combinations and can also be used to synthesize 2D halide perovskite superlattices through multiple repeated growth steps.

### Intragrain Perovskite Heterostructures

**Fig. 10:**
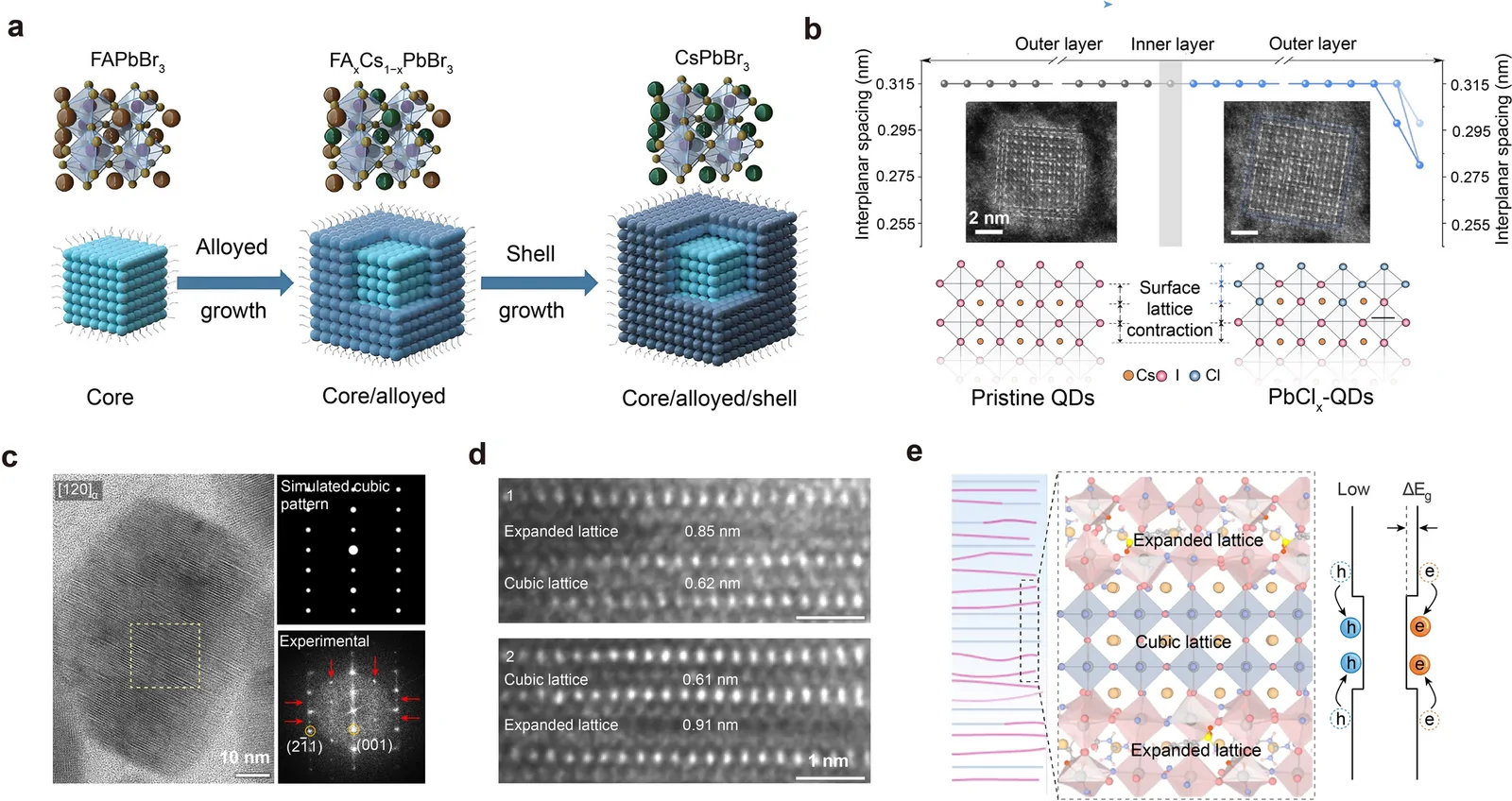
Intragrain perovskite heterostructures. **a** Schematics show the lattice structure and the synthesis process of FAPbBr_3_/CsPbBr_3_ core/shell PPHS [97]. Copyright 2020, Wiley–VCH.** b** TEM images of the position CsPbI_3_ QDs and PbCl_x_ covered QDs, the top side displays interplanar spacing as a function of layer position, and the bottom schematic illustrations of the PbCl_x_ modification on the surface crystal lattice [99]. Copyright 2024, Wiley–VCH. **c** TEM image of PTLA-inserted CsPbI_3-x_Br_x_ grain, the right side displays the simulated and experimental selected area electron diffraction patterns [100]. Copyright 2025, Springer Nature. **d** Magnified atomic-resolution TEM image of the CsPbI_3-x_Br_x_ intragrain perovskite heterostructure [100]. Copyright 2025, Springer Nature. **e** Schematic shows the lattice structure and carrier confinement effect of the intragrain heterostructure [100]. Copyright 2025, Springer Nature

Intragrain perovskite heterostructures refer to two or more different perovskite phases within a single grain. A typical example is the perovskite core/shell nanocrystal. Growing a robust shell on the core nanocrystal is one of the most well-known classical strategies for addressing surface defects and enhancing nanocrystal stability. Currently, perovskite core/shell nanocrystals with shell materials based on polymers [94], metal oxides [95], and other materials have been shown to have improved PLQY and environmental stability [96]. However, typically, easily preparable shells are insulating, which greatly hinders the application of such materials in electronic devices. Covering perovskite nanocrystals with another perovskite crystal shell and forming perovskite/perovskite core/shell heterostructures is an effective method to overcome electrical disadvantages. More importantly, using perovskite materials for the shell layer ensures perfect lattice matching, thereby achieving a more perfect core/shell interface. For example, Zhang et al. mixed PbBr_2_ and Cs_2_CO_3_, OA, and oleic acid with pre-synthesized FAPbBr_3_ NCs, resulting in the epitaxial growth of a uniform CsPbBr_3_ shell on the surface of FAPbBr_3_ nanocrystals [97]. By adjusting the molar ratio of Cs to FA, core/shell structures with different shell thicknesses can be produced. They proposed that an FA/Cs alloy transition layer first forms along the FAPbBr_3_ core, and, as the Cs content increases, a fully inorganic CsPbBr_3_ shell forms on the transition alloy layer (Fig. 10a). The confinement effect of the inorganic CsPbBr_3_ shell leads to a blue shift of the nanocrystals and increased exciton binding energy. Additionally, due to the reduced exciton–phonon coupling strength, the FWHM at room temperature decreases to 90 meV, and the PLQY reaches 93%. The PeLED based on the core/shell heterojunction achieves a peak EQE of 8.1%, which is approximately eight times higher than that of devices containing only the core.

Halide-heterogeneous core/shell perovskite nanocrystals have also been reported. Due to the significantly different ionic radii between I^−^ and Cl^−^, Cl is difficult to uniformly doped into the I lattice, which provides the prerequisite for achieving I-Cl heterostructures within the single grain and regulating the confinement properties as well as optical performance of the crystal. Based on the quantum confinement effect, to achieve emissions below 640 nm, the particle size of CsPbI_3_ QDs must be below approximately 5 nm [98]. However, ultra-small grain sizes and large specific surface areas increase the defect density in the grains. By introducing a CsPbCl_3_ shell onto the surface of CsPbI_3_ QDs to form a CsPbI_3_/CsPbCl_3_ core/shell confined system, it is possible to increase the grain size while confining excitons within a smaller range inside the grains, thereby reducing the emission wavelength and avoiding non-radiative recombination induced by surface defects. Following this principle, Feng et al. introduced Cl^−^ onto the surface of the synthesized CsPbI_3_ QDs by introducing acyl chloride after thermal injection [99]. Notably, since the overlying shell is sub-nanometer-thickness, they identified the shell layer as PbCl_x_ rather than CsPbCl_3_. Nevertheless, the shell features an interplanar spacing of 0.296 nm, approximately equal to that of the (200) planes in the CsPbCl_3_ phase, which demonstrates the essence of perovskite/perovskite heteroepitaxy (Fig. 10b). The average particle size of core/shell heterojunctions is 6.6 nm, with an emission peak at 633 nm and a PLQY close to unity. In contrast, the original 6.0 nm CsPbI_3_ QDs have a PL peak at 648 nm and a PLQY of 90%. These core/shell heterojunction films further enabled a highly efficient pure-red PeLED with a peak EQE of 26.1%. Furthermore, the core/shell heterojunction-based device showed an improvement in *T*_50_ of over 70 times compared to devices containing only the core.

In addition to core/shell heterojunctions, Song et al. recently demonstrated the construction of a heterostructure with alternating wide-bandgap and narrow-bandgap phases in 3D CsPbI_3-x_Br_x_ perovskite grains [100]. In this structure, the wide-bandgap regions exhibit an extended lattice constant due to the insertion of p-toluenesulfonyl-LAG (PTLA) organic molecules, while the narrow-bandgap regions retain the traditional 3D perovskite lattice (Fig. 10c, d). This grain-internal heterostructure differs from traditional 2D/3D PPHSs, since the organic molecules in wide-bandgap regions do not partition the inorganic layers but merely increase the lattice constant of the inorganic layers due to steric hindrance. This approach shows the advantage of retaining the excellent carrier transport properties of bulk perovskite. They also demonstrated that this unique intragrain quantum well structure reduces hole leakage from the perovskite layer to the electron transport layer in PeLEDs by enhancing carrier confinement, thereby mitigating efficiency roll-off caused by charge injection imbalance (Fig. 10e). Based on this, they demonstrated a pure-red PeLED with a peak EQE of 24.2%, a maximum luminance of 24,600 cd m^−2^, and a turn-on voltage of only 1.9 V. Additionally, due to the reduced ion migration in the intragrain heterostructure, the PeLED achieves an average *T*_50_ of 127 h at 100 cd m^−2^.

## Conclusions and Outlook

Metal halide perovskites have emerged as an ideal emissive layer for constructing the next generation of high-efficiency, high-color-purity, and low-cost LEDs. However, PeLEDs still face challenges such as non-radiative recombination induced by defect states and poor stability due to ion migration. PPHSs offer a breakthrough path for high-performance PeLEDs through bandgap engineering, defect passivation, charge confinement, and light management. As summarized in Table 1, PeLED devices with high efficiency (EQE > 30%) and high operational stability (*T*_50_ > 4000 h) have been demonstrated. In this review, we summarize PPHSs applied to PeLED devices, including vertical PPHSs, lateral PPHSs, and bulk PPHSs. We discuss the optoelectronic properties of different PPHSs and their mechanisms for enhancing the radiative recombination rate and operational stability. To further explore the potential of PPHSs in high-performance PeLEDs, key challenges and breakthrough directions should focus on interface carrier dynamics analysis, large-area controlled synthesis, and the development of advanced characterization technologies for PPHSs. Additionally, the unique structure and chemical space of PPHSs also offer opportunities for the development of new types of light-emitting devices (such as lasers and spin-polarized LEDs).

**Table 1:** Device performance summary of the LEDs based on PPHSs

Dimension structure	Spatial distribution	Chemical structure	EL peak (nm)	Peak EQE (%)	Peak luminance	Lifetime	Refs
3D/3D	Vertical	CsPbBr_3_/MAPbCl_3_	521	5.04	> 20,000 cd m^−2^	18.7 min (L_0_ = 2,150 cd m^−2^)	[26]
3D/3D	Bulk	CsCu_2_I_3_/Cs_3_Cu_2_I_5_	White	0.15	145 cd m^−2^	238.5 min (Under 7.0 V)	[59]
3D/3D	Bulk	α-CsPbI_3_/δ-CsPbI_3_	White	6.5	12,200 cd m^−2^	230 min (L_0_ = 100 cd m^−2^)	[62]
2D/3D	Vertical	p-FPEA_2_PbI_4_/CsPbI_x_Br_3-x_	674	22.2	> 1000 cd m^−2^	745 min (L_0_ = 100 cd m^−2^)	[66]
2D/3D	Vertical	(TeFBTT)_2_PbI_4_/CsPbI_x_Br_3-x_	798	31.4	929 W sr^−1^ m^−2^	/	[68]
2D/3D	Vertical	pFPEA_2_Pb(Br/Cl)_4_/CsPbBr_3-x_Cl_x_	478	12.3	802 cd m^−2^	8.2 min (L_0_ = 100 cd m^−2^)	[67]
2D/3D	Vertical	PEA_2_FA_n−1_Sn_n_I_3n+1_/FA_0.9_Cs_0.1_SnI_3_	898	11.6	89 W sr^−1^ m^−2^	23 min (Under 100 mA cm^−2^)	[70]
2D/3D	Bulk	CsPb_2_Br_5_/CsPbBr_3_	478	12.86	2311 cd m^−2^	14.2 min (L_0_ = 90 cd m^−2^)	[80]
0D/3D	Lateral	Cs_4_PbI_6_/FA_x_Cs_1-x_PbI_3_	705	21.2	1400 cd m^−2^	/	[82]
0D/3D	Bulk	CsPbI_3_ QDs/CsPb(Br_x_I_1-x_)_3_	~ 650	18	4700 cd m^−2^	2400 h (L_0_ = 100 cd m^−2^)	[85]
0D/3D	Bulk	CsPbI_3_ QDs/CsPb_1−x_Sr_x_Br_3_	495	13.8	> 6000 cd m^−2^	~ 1000 s (L_0_ = 440 cd m^−2^)	[32]
0D/3D	Bulk	Cs_4_PbBr_6_/CsPbBr_3_	522	17.0	> 200,000 cd m^−2^	25 min (L_0_ = 3000 cd m^−2^)	[86]
0D/3D	Bulk	Cs_4_PbBr_6_/CsPbBr_3_	518	28.2	> 150,000 cd m^−2^	4,291 h (L_0_ = 100 cd m^−2^)	[88]
0D/2D	Vertical	FAPbI_3_ QDs/5AVA_2_FA_n−1_Pb_n_I_3n+1_	770	21.82	> 10 W sr^−1^ m^−2^	117 min (L_0_ = 1 W sr ^–1^ m ^–2^)	[89]
0D/2D	Vertical	FAPbBr_3_ NCs/(OA)_2_(FA)_n-1_Pb_n_Br_3n+1_	~ 530	13.4	34,480 cd m^−2^	/	[90]
0D/2D	Bulk	CsPbI_3_ QDs/PEA_2_Cs_n−1_Pb_n_I_3n_	630	25.6	> 11,689 cd m^−2^	6330 min (L_0_ = 100 cd m^−2^)	[91]
Intragrain	Bulk	CsPbBr_3_/FAPbBr_3_	508	8.1	1758 cd m^−2^	47 min (L_0_ = 100 cd m^−2^)	[97]
Intragrain	Bulk	PbCl_x_/CsPbI_3_ QDs	638	26.1	2511 cd m^−2^	7.5 h (L_0_ = 100 cd m^−2^)	[99]
Intragrain	Bulk	PTLA-CsPbI_3-x_Br_x_/CsPbI_3-x_Br_x_	638	24.2	24,600 cd m^−2^	127 h (L_0_ = 100 cd m^−2^)	[100]

### Characterization Techniques Development of PPHSs

**Fig. 11:**
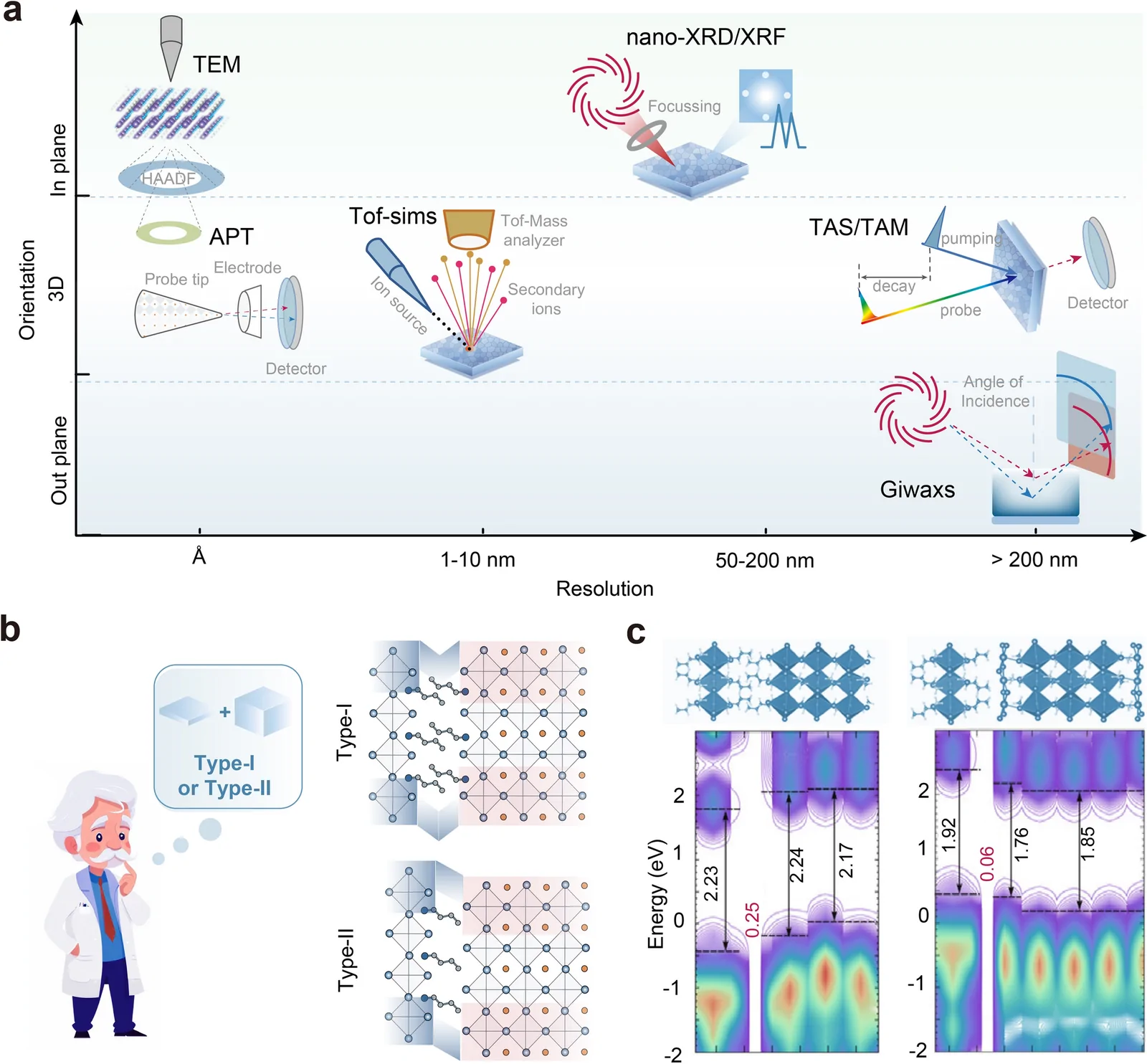
Characterization techniques and carrier dynamics investigation. **a** Representative microscopic characterization technique used for PPHS analysis. The horizontal axis shows the spatial resolution, and the vertical axis shows the detection dimension of these characterization techniques. **b** Schematic shows interfacial dipole moments and WF changes induced by different spatial arrangements of ligands in 2D/3D PPHS. **c** Band alignment at 2D/3D PPHS interface with single-layer and double-layer ligands, respectively [104]. Copyright 2019, American Chemical Society

The development of advanced microscopic characterization techniques is crucial for further understanding the carrier transport and recombination dynamics mechanisms in PPHSs and also guides the optimization of these PPHS-based optoelectronic devices. Over the past decade, significant progress has been made in understanding the optical and electrical properties of PPHSs through using advanced imaging characterization and ultrafast spectroscopy techniques (Fig. 11a). For perovskite heterojunctions at the nanoscale, achieving high-resolution imaging of chemical composition and crystal structure is critical. These techniques can map the local inhomogeneous properties of perovskite materials, laying the foundation for evaluating the optical and electrical properties associated with material structure and composition. For vertical PPHSs, out-of-plane directional microstructural and chemical composition characterization techniques, such as time-of-flight secondary ion mass spectrometry (TOF–SIMS) and grazing-incidence angle-dependent X-ray diffraction, can reflect the compositional and structural inhomogeneities of the material. For lateral PPHSs, in-plane directional characterization techniques are more critical, such as synchrotron-based nanoprobe imaging technology. Nano-XRD (X-ray diffraction), nano-XRF (X-ray fluorescence) and nano-FTIR (Fourier transform infrared spectroscopy) can achieve high-resolution in-plane composition and structural imaging by focusing the beam line to below tens of nanometers. However, imaging the chemical composition and structure of bulk PPHSs is more challenging. TEM is a commonly used atomic-resolution technique for characterizing microstructure and chemical composition, and it is the most effective method for reflecting composition distribution. Currently, combining FIB-TEM (focused ion beam TEM), STEM, and aberration correction techniques has enabled visualization of various perovskite heterojunctions ranging from a few nanometers to tens of nanometers. However, for some special heterostructures, such as core/shell QDs, three-dimensional, atomic-scale component analysis remains challenging, resulting in a lack of intuitive imaging of elemental distribution in previously reported perovskite core/shell heterojunctions. Combining three-dimensional atomic probe tomography is expected to provide atomic-scale imaging of the chemical composition and crystal structure of such ultra-small-sized heterojunctions [101].

### Carrier Dynamics Investigation of PPHSs

Further exploration is needed regarding the band alignment and carrier recombination dynamics at PPHS interfaces, particularly concerning carrier transport mechanisms at heterointerfaces, which remain poorly understood. Theoretically, type-I heterostructures enhance exciton radiative recombination, while type-II heterostructures enable efficient charge separation. Therefore, constructing highly efficient light-emitting diodes necessitates further investigation into band alignment, electron transport, and recombination mechanisms at PPHS interfaces. Taking 2D/3D PPHS as an example, the electronic band structure and carrier dynamics within heterojunctions are influenced by multiple factors, including the interface bonding configuration, thickness of inorganic layers and perovskite layers, orientation of the two perovskite phases, and surface termination at the interface.

Non-epitaxial PEA_2_PbBr_4_/CsPbBr_3_ PPHS with random interfaces has been demonstrated to exhibit type-I band alignment. However, Zhu et al. demonstrated that the band alignment between self-assembled PEA_2_PbBr_4_/CsPbBr_3_ epitaxial heterojunctions forms a type-II structure. For PEA_2_PbBr_4_/CsPbBr_3_ epitaxial heterojunctions, hole transfer occurs from CsPbBr_3_ to the adjacent PEA_2_PbBr_4_ layer, quenching CsPbBr_3_ emission. Consequently, PEA_2_PbBr_4_/CsPbBr_3_ epitaxial heterostructures exhibit excellent carrier separation properties but are disadvantageous for enhancing radiative recombination efficiency. Increasing the thickness of inorganic layers typically narrows the bandgap, causing the energy level alignment to shift from type-I to type-II alignment. For example, BA_2_MA_n-1_PbI_3n+1_ with *n* = 1 or 2 exhibits type-I alignment with MAPbI_3_, whereas *n* > 3 results in type-II alignment [102]. This implies that precise control over the thickness of two-dimensional layers within the heterostructure is necessary to suit different types of optoelectronic devices. Furthermore, Kuo et al. confirmed that when the 2D phase is oriented parallel to the 3D phase, the TRPL spectrum of CsPbBr_3_ exhibits an additional rise time, while the TRPL of PEA_2_PbBr_4_ shows attenuation [103]. This indicates energy transfer from the PEA_2_PbBr_4_ layer to CsPbBr_3_, corresponding to type-I band alignment. However, when the 2D perovskite layer thickness exceeds approximately 150 nm, the PL decay of CsPbBr_3_ accelerates significantly. This is attributed to the increased likelihood of excitons in the thicker 2D layer undergoing mutual recombination rather than transferring to the acceptor material. Furthermore, when the 2D phase is oriented perpendicular to the 3D phase, the energy transfer efficiency is significantly influenced by the spatial distance, meaning more transfer occurs at the 2D/3D phase interface. The form of surface termination at the heterointerface, such as the binding mode of ligands, has been shown to alter the interface band alignment and carrier transport/recombination mechanisms of the heterojunction by affecting the interface dipole. Bermudez et al. demonstrated that when both 2D and 3D perovskite phases exhibit complete surface capping with ligands, they exhibit conventional type-I band alignment [104]. However, at low ligand concentrations, the perovskite surface of the 2D phase is fully anchored by organic ligands, while the perovskite surface of the high *n*-value phase (3D phase) lacks ligands. The ligand-depleted 3D phase surface exhibits a net negative charge, generating an interfacial dipole moment directed from the 3D phase toward the 2D phase and ultimately forming a type-II band alignment (Fig. 11b, c). Overall, to construct PPHS with rapid energy transfer, efficient radiative recombination, and suitability for high-efficiency LED devices, systematic control is required over the heterojunction interface, the thickness of the inorganic layer or perovskite layer, and the orientation of the two-phase perovskite.

### Controlled Synthesis of PPHSs

Achieving PPHSs with well-defined structures and sharp interfaces is a prerequisite for enhancing their optoelectronic device performance. The synthesis of PPHS fundamentally involves precise control over the spatial distribution, crystal orientation, and interfacial chemistry of different perovskite phases [105]. Current mainstream preparation strategies primarily include sequential coating, epitaxial growth, self-assembly, and phase separation control. While these methods demonstrate unique advantages in constructing PPHS with diverse structures, they also face significant challenges in interface control, crystallization kinetics, and process compatibility.

**Fig. 12:**
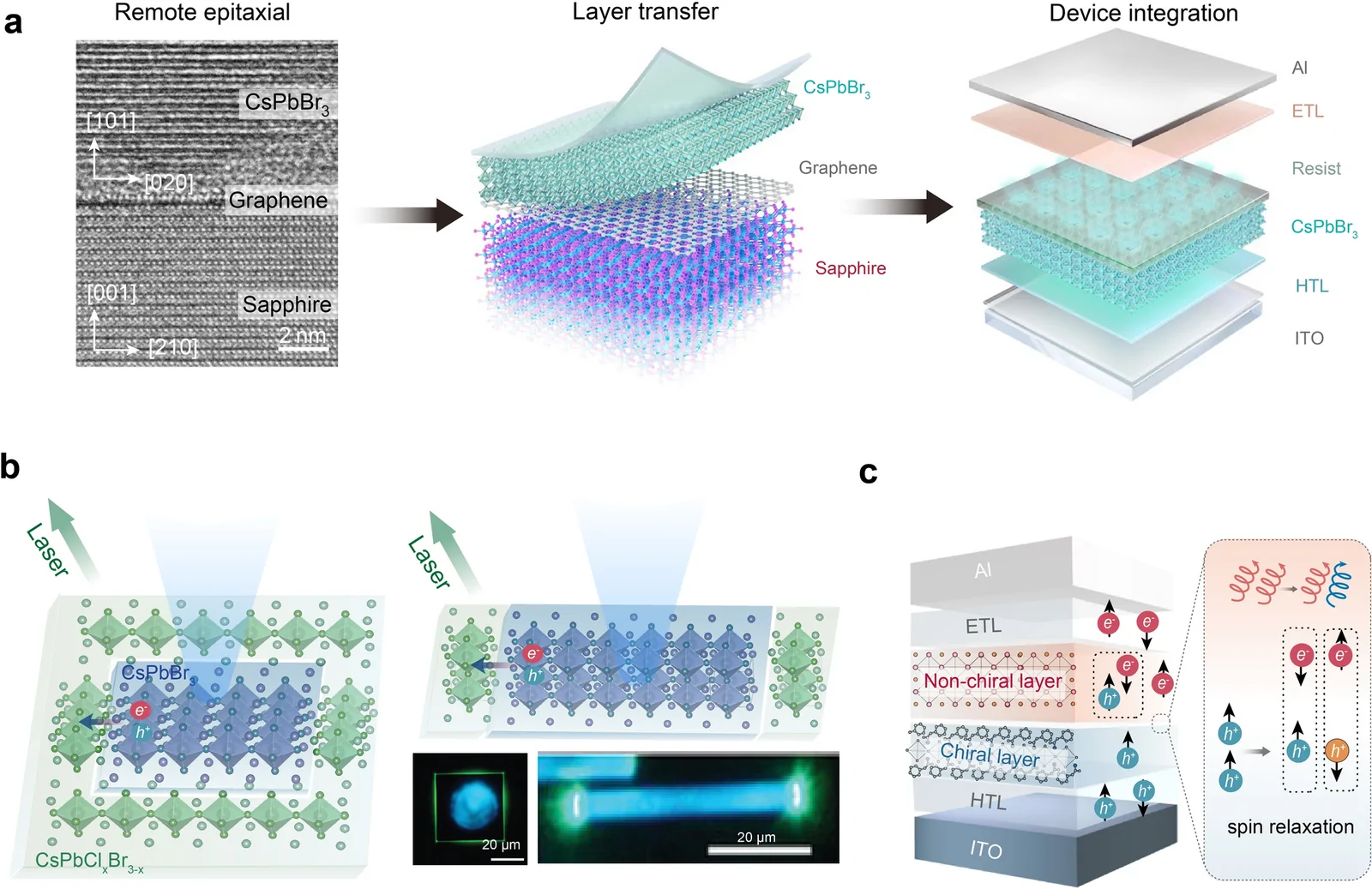
**a** Remote epitaxial, layer transfer, and device integration procedures of perovskite single-crystal films. Left: cross-sectional TEM image of sapphire/graphene/CsPbBr_3_ heterojunction. Middle: schematic shows the layer transfer process of CsPbBr_3_ single-crystal films. Right: schematic shows the structure of an integrated micro-PeLED device [107]. Copyright 2025, Springer Nature. **b** Schematic shows the structure of 2D and 1D CsPbCl_x_Br_(3−x)_/CsPbBr_3_ PPHSs and their laser beam pumping processes; insets display he fluorescent image of the PPHSs [110]. Copyright 2020, Wiley–VCH. **c** Schematic shows the structure of spin-polarized charge injection and circularly polarized EL emission in spin-polarized LEDs; inset shows the spin relaxation process

For constructing vertical PPHSs, sequential coating serves as the most intuitive approach, achieving vertical integration by successively depositing different perovskite precursors. This method offers simplicity, low cost, and compatibility with large-area solution processing techniques. However, its core challenge lies in preventing solvent erosion of the underlying layer and uncontrolled interfacial reactions during upper-layer deposition, which often leads to blurred interfaces and ion interdiffusion. To address this, strategies like using orthogonal solvents and introducing ultrathin interface modification layers have been widely adopted to achieve sharper, more stable heterointerfaces [106]. For applications requiring atomically sharp interfaces and superior crystal quality, epitaxial growth emerges as an ideal preparation route. Nevertheless, fabricating ultrathin heterojunction single crystals suitable for LED devices remains challenging. Furthermore, epitaxial growth’s incompatibility with current LED device integration processes poses a major barrier to its application. Recently, work by Yuan et al. demonstrated that large-area, single-crystal-grade, thickness-controllable perovskite epitaxial single-crystal films can be achieved by combining vapor deposition with remote epitaxy strategies [107]. By introducing a sub-nanometer graphene interlayer, they realized remote epitaxy and damage-free transfer of large-area, hundreds-of-nanometer-thick perovskite quasi-single-crystal films onto sapphire substrates, integrating them into high-efficiency micro-LEDs (Fig. 12a). This concept of remote epitaxy reduces lattice mismatch and interfacial stress at the epitaxial boundary, facilitating damage-free separation for film and device integration. Combining remote epitaxy with transfer printing technology opens possibilities for monolithic integration of multilayer perovskite films, demonstrating great promise for constructing high-efficiency optoelectronic devices such as tandem perovskite LEDs, tandem perovskite solar cells, and multiband detectors [108, 109].

The synthesis of lateral PPHSs hinges on achieving horizontal splicing of two distinct perovskite phases on a single substrate while maintaining sharp interfaces at the nanoscale or even atomic scale. Liquid-phase epitaxial growth emerges as an effective strategy. For non-monohalide PPHS, halide ion diffusion at the heterojunction interface poses a significant challenge. The core solution to this challenge involves employing large-volume, rigid π-conjugated organic ligands. These ligands stabilize the inorganic framework through strong intermolecular forces, thereby raising the energy barrier for halide ion migration [93]. Additionally, leveraging the sequential crystallization and lateral growth characteristics of different perovskite phases enables the self-assembly of horizontal PPHSs [82]. However, relevant reports remain scarce. Achieving controlled lateral growth of different perovskite phases on a substrate requires sequential nucleation and crystallization of distinct perovskites followed by out-of-plane diffusion growth. This poses significant challenges for crystallization kinetics control. To address this, real-time monitoring of assembly pathways using in situ characterization techniques is essential, coupled with precise regulation of kinetic parameters to establish a reliable process-to-structure mapping.

The most common methods for preparing bulk PPHSs utilize phase separation and self-assembly within the precursor solution to spontaneously form multiphase structures. This phase separation can be induced by precisely controlling precursor stoichiometry or by introducing different ligands to regulate the crystallization behavior of distinct phases, thereby controlling the final morphology and distribution of the two phases. The primary challenge in bulk heterojunction fabrication lies in avoiding macroscopic phase aggregation while achieving uniform, interpenetrating phase separation at the nanoscale, ensuring efficient charge injection and transport. Random or excessive phase separation can result in discontinuous charge transport pathways or carrier traps within a phase. On the other hand, to balance charge transport, the relative content and energy alignment of the two phases must be carefully regulated. Constructing bulk heterojunctions with epitaxial structures is a promising approach to enhance the recombination efficiency and stability of radiative recombination centers while maintaining good carrier transport capabilities. The success of epitaxy heavily relies on the rational design of ligand molecules and the control of the lattice constants of both phases. Achieving this goal necessitates establishing a perovskite database encompassing ligand structures and chemical compositions.

### Applications of PPHS in Emerging Light-Emitting Devices

Perovskite heterojunctions offer opportunities for the development of new types of light-emitting devices, such as high-performance lasers and electrically driven spin emission LEDs. Ren et al. demonstrated that 1D and 2D planar PPHSs can achieve low-threshold laser emission by combining energy funnels, optical waveguides, and photonic circulation effects [110]. They employed a two-step CVD growth strategy, demonstrating high-quality CsPbCl_x_Br_(3−x)_/CsPbBr_3_ PPHSs. The type-I band alignment enables energy transfer from the high-energy state of CsPbCl_x_Br_(3−x)_ to the low-energy state of CsPbBr_3_, which concentrates photo-generated carriers into the gain region, thereby promoting carrier concentration inversion. Furthermore, these planar heterojunctions feature an optical waveguide, tightly confining photons within the microcavity, and the edge/bilayer wide-bandgap phases act as mirrors, enabling the heterojunction microcrystal to behave as a total internal reflection-based whispering gallery mode (WGM) or Fabry–Perot (F–P) resonator. This resonant oscillation reduces the laser threshold, achieving laser emission with thresholds of approximately 3.5 µJ cm^−2^ and a Q factor of 3400 (Fig. 12b). PPHSs compose wide-bandgap chiral perovskite and narrow-bandgap achiral perovskites allow the delivery of room-temperature spin-LEDs. In spin-LEDs, circularly polarized light emission depends on the recombination of spin-polarized carriers. The spin-polarized carriers can be generated using chiral perovskites with chiral induction spin selectivity (CISS). For example, Kim et al. achieved the first room-temperature spin-LED with a spin resolution of 2.6% by introducing a chiral 2D (R-/S-MBA)_2_PbI_4_ perovskite layer between CsPbI_3_ nanocrystals and hole transport layer [111]. However, in this device structure, spin relaxation at the heterojunction interface between the chiral perovskite and non-chiral perovskite is a key factor affecting the spin discrimination. Although carriers passing through the chiral layer have been shown to have a polarization rate exceeding 80%, the majority of spin carriers undergo spin relaxation before recombination, resulting in the spin-LED’s low spin discrimination (Fig. 12c). Scattering relaxation caused by grain boundaries and interface defects is the primary relaxation pathway for carriers [112, 113]. Considering this, constructing a strict heteroepitaxial interface is a promising approach to reduce grain boundary scattering, enhance carrier spin relaxation lifetime, and improve the spin discrimination of spin-LEDs. Recently, Yao et al. employed an epitaxial welding strategy to fabricate self-assembled PPHSs composed of chiral 2D (R-MBA)_2_PbI_4_ and achiral CsPbBr_3_ nanocrystals in solution. In this PPHS with regularly aligned nanocrystals, they observed circularly polarized emission with enhanced circular polarization up to 10.3%. In contrast, no circularly polarized luminescence activity was observed in PPHS with randomly distributed nanocrystals. This result further demonstrates the superior chiral carrier transport properties of high-quality epitaxial interfaces [114].

## References

[1] B.R. Sutherland, E.H. Sargent, Perovskite photonic sources. Nat. Photonics **10**(5), 295–302 (2016). 10.1038/nphoton.2016.62

[2] T.-H. Han, K.Y. Jang, Y. Dong, R.H. Friend, E.H. Sargent et al., A roadmap for the commercialization of perovskite light emitters. Nat. Rev. Mater. **7**(10), 757–777 (2022). 10.1038/s41578-022-00459-4

[3] X.-K. Liu, W. Xu, S. Bai, Y. Jin, J. Wang et al., Metal halide perovskites for light-emitting diodes. Nat. Mater. **20**(1), 10–21 (2021). 10.1038/s41563-020-0784-7 32929252 10.1038/s41563-020-0784-7

[4] Y. Liu, Z. Ma, J. Zhang, Y. He, J. Dai et al., Light-emitting diodes based on metal halide perovskite and perovskite related nanocrystals. Adv. Mater. **37**(25), 2415606 (2025). 10.1002/adma.202415606 39887795 10.1002/adma.202415606 PMC12204139

[5] A.F. Gualdrón-Reyes, S. Masi, I. Mora-Sero, Progress in halide-perovskite nanocrystals with near-unity photoluminescence quantum yield. Trends Chem. **3**(6), 499–511 (2021). 10.1016/j.trechm.2021.03.005

[6] J. Chen, G.-P. Zhu, K.-L. Wang, C.-H. Chen, T.-Y. Teng et al., Unveiling full-dimensional distribution of trap states toward highly efficient perovskite photovoltaics. eScience **5**(2), 100326 (2025). 10.1016/j.esci.2024.100326

[7] N. Jiang, Z. Wang, Y. Zheng, Q. Guo, W. Niu et al., 2D/3D heterojunction perovskite light-emitting diodes with tunable ultrapure blue emissions. Nano Energy **97**, 107181 (2022). 10.1016/j.nanoen.2022.107181

[8] L. Protesescu, S. Yakunin, M.I. Bodnarchuk, F. Krieg, R. Caputo et al., Nanocrystals of cesium lead halide perovskites (CsPbX_3_, X = Cl, Br, and I): novel optoelectronic materials showing bright emission with wide color gamut. Nano Lett. **15**(6), 3692–3696 (2015). 10.1021/nl5048779 25633588 10.1021/nl5048779 PMC4462997

[9] Y.-H. Kim, J. Park, S. Kim, J.S. Kim, H. Xu et al., Exploiting the full advantages of colloidal perovskite nanocrystals for large-area efficient light-emitting diodes. Nat. Nanotechnol. **17**(6), 590–597 (2022). 10.1038/s41565-022-01113-4 35577974 10.1038/s41565-022-01113-4

[10] C. Sun, Y. Jiang, M. Cui, L. Qiao, J. Wei et al., High-performance large-area quasi-2D perovskite light-emitting diodes. Nat. Commun. **12**, 2207 (2021). 10.1038/s41467-021-22529-x 33850141 10.1038/s41467-021-22529-x PMC8044177

[11] M. Li, Y. Yang, Z. Kuang, C. Hao, S. Wang et al., Acceleration of radiative recombination for efficient perovskite LEDs. Nature **630**(8017), 631–635 (2024). 10.1038/s41586-024-07460-7 38811739 10.1038/s41586-024-07460-7 PMC11186751

[12] W. Bai, T. Xuan, H. Zhao, H. Dong, X. Cheng et al., Perovskite light-emitting diodes with an external quantum efficiency exceeding 30. Adv. Mater. **35**(39), e2302283 (2023). 10.1002/adma.202302283 37246938 10.1002/adma.202302283

[13] J. Jiang, Z. Xia, M. Shi, Z. Yin, W. Huang et al., Efficient red perovskite LEDs with iodine management *via* volatile additive I_2_. Adv. Mater. **37**(32), 2503699 (2025). 10.1002/adma.202503699 10.1002/adma.202503699 40405631

[14] C. Peng, H. Yao, O. Ali, W. Chen, Y. Yang et al., Weakly space-confined all-inorganic perovskites for light-emitting diodes. Nature **643**(8070), 96–103 (2025). 10.1038/s41586-025-09137-1 40500444 10.1038/s41586-025-09137-1

[15] C. Li, K.P. Loh, K. Leng, Organic-inorganic hybrid perovskites and their heterostructures. Matter **5**(12), 4153–4169 (2022). 10.1016/j.matt.2022.11.002

[16] X. Cheng, Y. Han, B.-B. Cui, Fabrication strategies and optoelectronic applications of perovskite heterostructures. Adv. Opt. Mater. **10**(5), 2102224 (2022). 10.1002/adom.202102224

[17] Z. Zhang, S. Wang, X. Liu, Y. Chen, C. Su et al., Metal halide perovskite/2D material heterostructures: syntheses and applications. Small Methods **5**(4), 2000937 (2021). 10.1002/smtd.202000937 10.1002/smtd.202000937 34927847

[18] M. Wang, Z. Wan, X. Meng, Z. Li, X. Ding et al., Heterostructured Co/Mo-sulfide catalyst enables unbiased solar water splitting by integration with perovskite solar cells. Appl. Catal. B Environ. **309**, 121272 (2022). 10.1016/j.apcatb.2022.121272

[19] Y. Wang, T. Wu, J. Barbaud, W. Kong, D. Cui et al., Stabilizing heterostructures of soft perovskite semiconductors. Science **365**(6454), 687–691 (2019). 10.1126/science.aax8018 31416961 10.1126/science.aax8018

[20] Z. Dong, Z. Zhang, Y. Jiang, Y. Chu, J. Xu, Embedding CsPbBr_3_ perovskite quantum dots into mesoporous Ti_O_2 beads as an S-scheme heterojunction for C_O_2 photoreduction. Chem. Eng. J. **433**, 133762 (2022). 10.1016/j.cej.2021.133762

[21] D. Liu, T.L. Kelly, Perovskite solar cells with a planar heterojunction structure prepared using room-temperature solution processing techniques. Nat. Photonics **8**(2), 133–138 (2014). 10.1038/nphoton.2013.342

[22] S. Chander, S.K. Tripathi, Recent advancement in efficient metal oxide-based flexible perovskite solar cells: a short review. Mater. Adv. **3**(19), 7198–7211 (2022). 10.1039/D2MA00700B

[23] T. Wang, W. Deng, J. Cao, F. Yan, Recent progress on heterojunction engineering in perovskite solar cells. Adv. Energy Mater. **13**(33), 2201436 (2023). 10.1002/aenm.202201436

[24] X. Wu, B. Li, Z. Zhu, C.-C. Chueh, A.K.Y. Jen, Designs from single junctions, heterojunctions to multijunctions for high-performance perovskite solar cells. Chem. Soc. Rev. **50**(23), 13090–13128 (2021). 10.1039/D1CS00841B 34676850 10.1039/d1cs00841b

[25] B.M. Wieliczka, S.N. Habisreutinger, K. Schutt, J.L. Blackburn, J.M. Luther, Nanocrystal-enabled perovskite heterojunctions in photovoltaic applications and beyond. Adv. Energy Mater. **13**(22), 2204351 (2023). 10.1002/aenm.202204351

[26] D.-H. Kang, S.-G. Kim, Y.C. Kim, I.T. Han, H.J. Jang et al., CsPbBr_3_/CH_3_NH_3_PbCl_3_ double layer enhances efficiency and lifetime of perovskite light-emitting diodes. ACS Energy Lett. **5**(7), 2191–2199 (2020). 10.1021/acsenergylett.0c01036

[27] J. Wang, L. Bi, X. Huang, Q. Feng, M. Liu et al., Bilayer interface engineering through 2D/3D perovskite and surface dipole for inverted perovskite solar modules. eScience **4**(6), 100308 (2024). 10.1016/j.esci.2024.100308

[28] Y. Wang, Z. Chen, F. Deschler, X. Sun, T.-M. Lu et al., Epitaxial halide perovskite lateral double heterostructure. ACS Nano **11**(3), 3355–3364 (2017). 10.1021/acsnano.7b00724 28245110 10.1021/acsnano.7b00724

[29] P. Du, J. Li, L. Wang, L. Sun, X. Wang et al., Efficient and large-area all vacuum-deposited perovskite light-emitting diodes *via* spatial confinement. Nat. Commun. **12**(1), 4751 (2021). 10.1038/s41467-021-25093-6 34362915 10.1038/s41467-021-25093-6 PMC8346511

[30] X. Li, W. Ma, D. Liang, W. Cai, S. Zhao et al., High-performance CsPbBr_3_@Cs_4_PbBr_6_/SiO_2_ nanocrystals *via* double coating layers for white light emission and visible light communication. eScience **2**(6), 646–654 (2022). 10.1016/j.esci.2022.10.005

[31] P. Liu, Y. Xu, B. Li, Y. Zhang, H. Lian et al., Structural engineering of BaWO_4_/CsPbX_3_/CsPb_2_X_5_ (X = Cl, Br, I) heterostructures towards ultrastable and tunable photoluminescence. Nano Res. **17**(3), 1636–1645 (2024). 10.1007/s12274-023-5922-5

[32] Y. Liu, Z. Li, J. Xu, Y. Dong, B. Chen et al., Wide-bandgap perovskite quantum dots in perovskite matrix for sky-blue light-emitting diodes. J. Am. Chem. Soc. **144**(9), 4009–4016 (2022). 10.1021/jacs.1c12556 35192324 10.1021/jacs.1c12556

[33] P.C. Sercel, J.L. Lyons, D. Wickramaratne, R. Vaxenburg, N. Bernstein et al., Exciton fine structure in perovskite nanocrystals. Nano Lett. **19**(6), 4068–4077 (2019). 10.1021/acs.nanolett.9b01467 31088061 10.1021/acs.nanolett.9b01467

[34] P. Pang, Z. Xing, J. Xia, B. Wang, Z. Zhang et al., Deep-blue light-emitting diodes constructed with perovskite quasi-2D and nanocrystal mixtures. Adv. Opt. Mater. **10**(20), 2201112 (2022). 10.1002/adom.202201112

[35] Z. Xing, G. Jin, Q. Du, P. Pang, T. Liu et al., Ions-induced assembly of perovskite nanocomposites for highly efficient light-emitting diodes with EQE exceeding 30%. Adv. Mater. **36**(46), 2406706 (2024). 10.1002/adma.202406706 10.1002/adma.202406706 39308291

[36] Q. Zhang, D. Zhang, Z. Liao, Y.B. Cao, M. Kumar et al., Perovskite light-emitting diodes with quantum wires and nanorods. Adv. Mater. **37**(23), 2405418 (2025). 10.1002/adma.202405418 39183527 10.1002/adma.202405418 PMC12160700

[37] L. Lu, T. Zheng, Q. Wu, A.M. Schneider, D. Zhao et al., Recent advances in bulk heterojunction polymer solar cells. Chem. Rev. **115**(23), 12666–12731 (2015). 10.1021/acs.chemrev.5b00098 26252903 10.1021/acs.chemrev.5b00098

[38] Z. Zhou, H.W. Qiao, Y. Hou, H.G. Yang, S. Yang, Epitaxial halide perovskite-based materials for photoelectric energy conversion. Energy Environ. Sci. **14**(1), 127–157 (2021). 10.1039/d0ee02902e

[39] J.Y. Park, Y.H. Lee, M.A. Zaman Mamun, M.M. Fahimul Islam, S. Zhang et al., Electrically induced directional ion migration in two-dimensional perovskite heterostructures. Matter **7**(5), 1817–1832 (2024). 10.1016/j.matt.2024.03.005

[40] C.P. Clark, J.E. Mann, J.S. Bangsund, W.-J. Hsu, E.S. Aydil et al., Formation of stable metal halide perovskite/perovskite heterojunctions. ACS Energy Lett. **5**(11), 3443–3451 (2020). 10.1021/acsenergylett.0c01609

[41] W. Zhu, S. Wang, X. Zhang, A. Wang, C. Wu et al., Ion migration in organic–inorganic hybrid perovskite solar cells: current understanding and perspectives. Small **18**(15), 2105783 (2022). 10.1002/smll.202105783 10.1002/smll.202105783 35038213

[42] N. Li, Y. Jia, Y. Guo, N. Zhao, Ion migration in perovskite light-emitting diodes: mechanism, characterizations, and material and device engineering. Adv. Mater. **34**(19), e2108102 (2022). 10.1002/adma.202108102 34847262 10.1002/adma.202108102

[43] L. Zhang, Y. Jiang, Y. Feng, M. Cui, S. Li et al., Manipulating local lattice distortion for spectrally stable and efficient mixed-halide blue perovskite LEDs. Angew. Chem. Int. Ed. **62**(21), e202302184 (2023). 10.1002/anie.202302184 10.1002/anie.202302184 36866612

[44] G. Sarkar, P. Deswal, D. Ghosh, Ion diffusion dynamics and halogen mixing at the heterojunction of halide perovskites: atomistic insights. J. Phys. Chem. C **128**(4), 1762–1772 (2024). 10.1021/acs.jpcc.3c06329

[45] D.-J. Xue, Y. Hou, S.-C. Liu, M. Wei, B. Chen et al., Regulating strain in perovskite thin films through charge-transport layers. Nat. Commun. **11**, 1514 (2020). 10.1038/s41467-020-15338-1 32251277 10.1038/s41467-020-15338-1 PMC7090003

[46] M.P. Hautzinger, E.K. Raulerson, S.P. Harvey, T. Liu, D. Duke et al., Metal halide perovskite heterostructures: blocking anion diffusion with single-layer graphene. J. Am. Chem. Soc. **145**(4), 2052–2057 (2023). 10.1021/jacs.2c12441 36649211 10.1021/jacs.2c12441 PMC9896553

[47] S.-H. Chin, L. Mardegan, F. Palazon, M. Sessolo, H.J. Bolink, Dimensionality controls anion intermixing in electroluminescent perovskite heterojunctions. ACS Photonics **9**(7), 2483–2488 (2022). 10.1021/acsphotonics.2c00604 35880074 10.1021/acsphotonics.2c00604 PMC9305999

[48] Z. Hu, X. Liu, P.L. Hernández-Martínez, S. Zhang, P. Gu et al., Interfacial charge and energy transfer in van der Waals heterojunctions. InfoMat **4**(3), e12290 (2022). 10.1002/inf2.12290

[49] C. Lin, Y. Tang, W. Xu, P. Kumar, L. Dou, Charge transfer in 2D halide perovskites and 2D/3D heterostructures. ACS Energy Lett. **9**(8), 3877–3886 (2024). 10.1021/acsenergylett.4c01530

[50] Z. Han, Y. Song, Y. Jia, Y. Wang, J. Shi et al., Classification and characterization methods for heterojunctions. Adv. Mater. Interfaces **12**(15), 2500191 (2025). 10.1002/admi.202500191

[51] J.T. DuBose, P.V. Kamat, Energy versus electron transfer: managing excited-state interactions in perovskite nanocrystal–molecular hybrids. Chem. Rev. **122**(15), 12475–12494 (2022). 10.1021/acs.chemrev.2c00172 35793168 10.1021/acs.chemrev.2c00172

[52] L. Lei, D. Seyitliyev, S. Stuard, J. Mendes, Q. Dong et al., Efficient energy funneling in quasi-2D perovskites: from light emission to lasing. Adv. Mater. **32**(16), 1906571 (2020). 10.1002/adma.201906571 10.1002/adma.201906571 32108964

[53] Y. Song, C. Zhang, W. Liu, X. Li, H. Long et al., High-efficiency energy transfer in perovskite heterostructures. Opt. Express **26**(14), 18448–18456 (2018). 10.1364/OE.26.018448 30114024 10.1364/OE.26.018448

[54] X. Chen, P.V. Kamat, C. Janáky, G.F. Samu, Charge transfer kinetics in halide perovskites: on the constraints of time-resolved spectroscopy measurements. ACS Energy Lett. **9**(6), 3187–3203 (2024). 10.1021/acsenergylett.4c00736 38911533 10.1021/acsenergylett.4c00736 PMC11190987

[55] S. Deng, J.M. Snaider, Y. Gao, E. Shi, L. Jin et al., Long-lived charge separation in two-dimensional ligand-perovskite heterostructures. J. Chem. Phys. **152**(4), 044711 (2020). 10.1063/1.5131801 32007060 10.1063/1.5131801

[56] N.T. Shewmon, H. Yu, I. Constantinou, E. Klump, F. So, Formation of perovskite heterostructures by ion exchange. ACS Appl. Mater. Interfaces **8**(48), 33273–33279 (2016). 10.1021/acsami.6b10034 27934163 10.1021/acsami.6b10034

[57] Y. Lei, Y. Chen, R. Zhang, Y. Li, Q. Yan et al., A fabrication process for flexible single-crystal perovskite devices. Nature **583**(7818), 790–795 (2020). 10.1038/s41586-020-2526-z 32728239 10.1038/s41586-020-2526-z

[58] L. Ye, Y. Gao, Y. Feng, X. Zhu, Z. Ma et al., Suppressing interlayer ion migration in CsPbX_3_ nanocrystal films for realizing efficient and stable electroluminescence. Adv. Mater. **37**(33), 2505214 (2025). 10.1002/adma.202505214 10.1002/adma.202505214 40444367

[59] Z. Ma, Z. Shi, D. Yang, Y. Li, F. Zhang et al., High color-rendering index and stable white light-emitting diodes by assembling two broadband emissive self-trapped excitons. Adv. Mater. **33**(2), 2001367 (2021). 10.1002/adma.202001367 10.1002/adma.202001367 33225543

[60] B. Zhang, X. Wu, S. Zhou, G. Liang, Q. Hu, Self-trapped exciton emission in inorganic copper(I) metal halides. Front. Optoelectron. **14**(4), 459–472 (2021). 10.1007/s12200-021-1133-4 36637760 10.1007/s12200-021-1133-4 PMC9743870

[61] W. Zhang, Z. Chu, J. Jiang, S. Zhang, H. Hao et al., High-performance white emission from Cu-based perovskite compound. Adv. Opt. Mater. **12**(18), 2400092 (2024). 10.1002/adom.202400092

[62] J. Chen, J. Wang, X. Xu, J. Li, J. Song et al., Efficient and bright white light-emitting diodes based on single-layer heterophase halide perovskites. Nat. Photonics **15**(3), 238–244 (2021). 10.1038/s41566-020-00743-1

[63] Z. Xiao, R.A. Kerner, L. Zhao, N.L. Tran, K.M. Lee et al., Efficient perovskite light-emitting diodes featuring nanometre-sized crystallites. Nat. Photonics **11**(2), 108–115 (2017). 10.1038/nphoton.2016.269

[64] J.S. Kim, J.-M. Heo, G.-S. Park, S.-J. Woo, C. Cho et al., Ultra-bright, efficient and stable perovskite light-emitting diodes. Nature **611**(7937), 688–694 (2022). 10.1038/s41586-022-05304-w 36352223 10.1038/s41586-022-05304-w

[65] L. Zhang, C. Sun, T. He, Y. Jiang, J. Wei et al., High-performance quasi-2D perovskite light-emitting diodes: from materials to devices. Light Sci. Appl. **10**(1), 61 (2021). 10.1038/s41377-021-00501-0 33741895 10.1038/s41377-021-00501-0 PMC7979804

[66] D. Zhang, J. Liu, X. Duan, K. Chen, B. Yu et al., Efficient deep-red perovskite light-emitting diodes based on a vertical 3D/2D perovskite heterojunction. Adv. Funct. Mater. **34**(39), 2403874 (2024). 10.1002/adfm.202403874

[67] K. Zhang, Z. Su, Y. Shen, L.-X. Cao, X.-Y. Zeng et al., Top-down exfoliation process constructing 2D/3D heterojunction toward ultrapure blue perovskite light-emitting diodes. ACS Nano **18**(5), 4570–4578 (2024). 10.1021/acsnano.3c12433 38277481 10.1021/acsnano.3c12433

[68] S.-D. Baek, W. Shao, W. Feng, Y. Tang, Y.H. Lee et al., Grain engineering for efficient near-infrared perovskite light-emitting diodes. Nat. Commun. **15**, 10760 (2024). 10.1038/s41467-024-55075-3 39737972 10.1038/s41467-024-55075-3 PMC11685452

[69] Z. Zhu, C. Zhu, L. Yang, Q. Chen, L. Zhang et al., Room-temperature epitaxial welding of 3D and 2D perovskites. Nat. Mater. **21**(9), 1042–1049 (2022). 10.1038/s41563-022-01311-4 35879439 10.1038/s41563-022-01311-4

[70] H. Min, N. Wang, N. Chen, Y. Tong, Y. Wang et al., Spin coating epitaxial heterodimensional tin perovskites for light-emitting diodes. Nat. Nanotechnol. **19**(5), 632–637 (2024). 10.1038/s41565-023-01588-9 38216685 10.1038/s41565-023-01588-9

[71] S. Ye, Y. Chen, H. Wu, H. Lang, H. Wang et al., Heterodimensional epitaxy of CsSnI_3_ microcrystalline cubes for bright and efficient near-infrared light-emitting diode. Adv. Mater. (2025). 10.1002/adma.202512927 10.1002/adma.202512927 41178151

[72] Z. Wang, Q. Lin, F.P. Chmiel, N. Sakai, L.M. Herz et al., Efficient ambient-air-stable solar cells with 2D–3D heterostructured butylammonium-caesium-formamidinium lead halide perovskites. Nat. Energy **2**, 17135 (2017). 10.1038/nenergy.2017.135

[73] C. Luo, G. Zheng, F. Gao, X. Wang, Y. Zhao et al., Facet orientation tailoring *via* 2D-seed- induced growth enables highly efficient and stable perovskite solar cells. Joule **6**(1), 240–257 (2022). 10.1016/j.joule.2021.12.006

[74] I. Dursun, M. De Bastiani, B. Turedi, B. Alamer, A. Shkurenko et al., CsPb_2_Br_5_ single crystals: synthesis and characterization. Chemsuschem **10**(19), 3746–3749 (2017). 10.1002/cssc.201701131 28766308 10.1002/cssc.201701131

[75] X. Zhang, B. Xu, J. Zhang, Y. Gao, Y. Zheng et al., All-inorganic perovskite nanocrystals for high-efficiency light emitting diodes: dual-phase CsPbBr_3_-CsP_b_2B_r_5 composites. Adv. Funct. Mater. **26**(25), 4595–4600 (2016). 10.1002/adfm.201600958

[76] J. Lv, L. Fang, J. Shen, Synthesis of highly luminescent CsPb_2_Br_5_ nanoplatelets and their application for light-emitting diodes. Mater. Lett. **211**, 199–202 (2018). 10.1016/j.matlet.2017.09.106

[77] S.K. Balakrishnan, P.V. Kamat, Ligand assisted transformation of cubic CsPbBr_3_ nanocrystals into two-dimensional CsPb_2_Br_5_ nanosheets. Chem. Mater. **30**(1), 74–78 (2018). 10.1021/acs.chemmater.7b04142

[78] B.-S. Zhu, H.-Z. Li, J. Ge, H.-D. Li, Y.-C. Yin et al., Room temperature precipitated dual phase CsPbBr_3_–CsPb_2_Br_5_ nanocrystals for stable perovskite light emitting diodes. Nanoscale **10**(41), 19262–19271 (2018). 10.1039/C8NR06879H 30324957 10.1039/c8nr06879h

[79] L. Wang, D. Ma, C. Guo, X. Jiang, M. Li et al., CsPbBr_3_ nanocrystals prepared by high energy ball milling in one-step and structural transformation from CsPbBr_3_ to CsPb_2_Br_5_. Appl. Surf. Sci. **543**, 148782 (2021). 10.1016/j.apsusc.2020.148782

[80] Y. Wang, S. Jin, S. Jiang, S. Zhai, L. Liu et al., CsPb_2_Br_5_ plates/quasi-2D perovskite heterojunction for efficient sky-blue light-emitting diodes. ACS Appl. Mater. Interfaces **16**(42), 57355–57364 (2024). 10.1021/acsami.4c11568 39382093 10.1021/acsami.4c11568

[81] C. Abia, C.A. López, J. Gainza, J.E.F.S. Rodrigues, B. Fragoso et al., Structural features and optical properties of all-inorganic zero-dimensional halides Cs_4_PbBr_6__*–*_xIx obtained by mechanochemistry. ACS Appl. Mater. Interfaces **15**(34), 40762–40771 (2023). 10.1021/acsami.3c07707 37595125 10.1021/acsami.3c07707 PMC10472433

[82] Y. Ke, J. Guo, D. Kong, J. Wang, G. Kusch et al., Efficient and bright deep-red light-emitting diodes based on a lateral 0D/3D perovskite heterostructure. Adv. Mater. **36**(20), e2207301 (2024). 10.1002/adma.202207301 36524445 10.1002/adma.202207301

[83] O. Chen, J. Zhao, V.P. Chauhan, J. Cui, C. Wong et al., Compact high-quality CdSe–CdS core–shell nanocrystals with narrow emission linewidths and suppressed blinking. Nat. Mater. **12**(5), 445–451 (2013). 10.1038/nmat3539 23377294 10.1038/nmat3539 PMC3677691

[84] Z. Ning, X. Gong, R. Comin, G. Walters, F. Fan et al., Quantum-dot-in-perovskite solids. Nature **523**(7560), 324–328 (2015). 10.1038/nature14563 26178963 10.1038/nature14563

[85] Y. Liu, Y. Dong, T. Zhu, D. Ma, A. Proppe et al., Bright and stable light-emitting diodes based on perovskite quantum dots in perovskite matrix. J. Am. Chem. Soc. **143**(38), 15606–15615 (2021). 10.1021/jacs.1c02148 34542273 10.1021/jacs.1c02148

[86] X. Gong, X. Hao, J. Si, Y. Deng, K. An et al., High-performance all-inorganic architecture perovskite light-emitting diodes based on tens-of-nanometers-sized CsPbBr_3_ emitters in a carrier-confined heterostructure. ACS Nano **18**(12), 8673–8682 (2024). 10.1021/acsnano.3c09004 38471123 10.1021/acsnano.3c09004

[87] N. Meng, Y. Li, X. Shi, Z. Wang, J. Liu et al., Fully thermal-evaporated perovskite light-emitting diodes with brightness exceeding 240 000 nits. Adv. Funct. Mater. (2025). 10.1002/adfm.202510484

[88] Z. Xia, J. Jiang, A. Wang, D. An, Z. Li et al., Overall performance improvement of perovskite green LEDs by CsPbBr_3_&Cs_4_PbBr_6_ nanocrystals and molecular doping. Adv. Mater. **37**(34), 2506187 (2025). 10.1002/adma.202506187 10.1002/adma.202506187 40489126

[89] H.-H. Li, Y. Wang, Z.-S. Liu, F. Zhao, Y.-K. Wang et al., Hybrid-dimensional heterostructure enables efficient near-infrared perovskite quantum dot light-emitting diodes. ACS Nano **19**(28), 25930–25938 (2025). 10.1021/acsnano.5c05939 40637111 10.1021/acsnano.5c05939

[90] X.Y. Chin, A. Perumal, A. Bruno, N. Yantara, S.A. Veldhuis et al., Self-assembled hierarchical nanostructured perovskites enable highly efficient LEDs *via* an energy cascade. Energy Environ. Sci. **11**(7), 1770–1778 (2018). 10.1039/C8EE00293B

[91] K. Wei, T. Zhou, Y. Jiang, C. Sun, Y. Liu et al., Perovskite heteroepitaxy for high-efficiency and stable pure-red LEDs. Nature **638**(8052), 949–956 (2025). 10.1038/s41586-024-08503-9 39972133 10.1038/s41586-024-08503-9

[92] M. Xia, T. Wang, Y. Lu, Y. Li, B. Li et al., Kinetic Wulff-shaped heteroepitaxy of phase-pure 2D perovskite heterostructures with deterministic slab thickness. Nat. Synth. **4**(3), 380–390 (2025). 10.1038/s44160-024-00692-5

[93] E. Shi, B. Yuan, S.B. Shiring, Y. Gao, Akriti et al., Two-dimensional halide perovskite lateral epitaxial heterostructures. Nature **580**(7805), 614–620 (2020). 10.1038/s41586-020-2219-7 32350477 10.1038/s41586-020-2219-7

[94] B. Zhao, S. Bai, V. Kim, R. Lamboll, R. Shivanna et al., High-efficiency perovskite–polymer bulk heterostructure light-emitting diodes. Nat. Photonics **12**(12), 783–789 (2018). 10.1038/s41566-018-0283-4

[95] C. Rossi, R. Scarfiello, R. Brescia, L. Goldoni, G. Caputo et al., Exploiting the transformative features of metal halides for the synthesis of CsPbBr_3_@SiO_2_ core–shell nanocrystals. Chem. Mater. **34**(1), 405–413 (2022). 10.1021/acs.chemmater.1c03749

[96] G.H. Ahmed, J. Yin, O.M. Bakr, O.F. Mohammed, Successes and challenges of core/shell lead halide perovskite nanocrystals. ACS Energy Lett. **6**(4), 1340–1357 (2021). 10.1021/acsenergylett.1c00076

[97] C. Zhang, S. Wang, X. Li, M. Yuan, L. Turyanska et al., Core/shell perovskite nanocrystals: synthesis of highly efficient and environmentally stable FAPbBr_3_/CsPbBr_3_ for LED applications. Adv. Funct. Mater. **30**(31), 1910582 (2020). 10.1002/adfm.201910582

[98] J. Ye, D. Gaur, C. Mi, Z. Chen, I.L. Fernández et al., Strongly-confined colloidal lead-halide perovskite quantum dots: from synthesis to applications. Chem. Soc. Rev. **53**(16), 8095–8122 (2024). 10.1039/d4cs00077c 38894687 10.1039/d4cs00077c

[99] Y. Feng, H. Li, M. Zhu, Y. Gao, Q. Cai et al., Nucleophilic reaction-enabled chloride modification on CsPbI3 quantum dots for pure red light-emitting diodes with efficiency exceeding 26%. Angew. Chem. Int. Ed. **63**(11), e202318777 (2024). 10.1002/anie.202318777 10.1002/anie.202318777 38258990

[100] Y.-H. Song, B. Li, Z.-J. Wang, X.-L. Tai, G.-J. Ding et al., Intragrain 3D perovskite heterostructure for high-performance pure-red perovskite LEDs. Nature **641**(8062), 352–357 (2025). 10.1038/s41586-025-08867-6 40335712 10.1038/s41586-025-08867-6

[101] W. Xu, J. Liu, B. Dong, J. Huang, H. Shi et al., Atomic-scale imaging of ytterbium ions in lead halide perovskites. Sci. Adv. **9**(35), eadi7931 (2023). 10.1126/sciadv.adi7931 37656785 10.1126/sciadv.adi7931 PMC10854428

[102] S. Sidhik, Y. Wang, M. De Siena, R. Asadpour, A.J. Torma et al., Deterministic fabrication of 3D/2D perovskite bilayer stacks for durable and efficient solar cells. Science **377**(6613), 1425–1430 (2022). 10.1126/science.abq7652 36137050 10.1126/science.abq7652

[103] M.-Y. Kuo, N. Spitha, M.P. Hautzinger, P.-L. Hsieh, J. Li et al., Distinct carrier transport properties across horizontally vs vertically oriented heterostructures of 2D/3D perovskites. J. Am. Chem. Soc. **143**(13), 4969–4978 (2021). 10.1021/jacs.0c10000 33764051 10.1021/jacs.0c10000

[104] R. Quintero-Bermudez, A.H. Proppe, A. Mahata, P. Todorović́, S.O. Kelley et al., Ligand-induced surface charge density modulation generates local type-II band alignment in reduced-dimensional perovskites. J. Am. Chem. Soc. **141**(34), 13459–13467 (2019). 10.1021/jacs.9b04801 31366193 10.1021/jacs.9b04801

[105] X. Xu, X. Wang, Perovskite nano-heterojunctions: synthesis, structures, properties, challenges, and prospects. Small Struct. **1**(1), 2000009 (2020). 10.1002/sstr.202000009

[106] X. Huang, Q. Xiong, Z. Su, J. Zhou, F. Cao et al., Orthogonal solvent approach in dimensionality-heterointerface perovskite photovoltaics. ACS Energy Lett. **10**(2), 982–990 (2025). 10.1021/acsenergylett.4c03263

[107] M. Yuan, J. Feng, H. Li, H. Gao, Y. Qiu et al., Remote epitaxial crystalline perovskites for ultrahigh-resolution micro-LED displays. Nat. Nanotechnol. **20**(3), 381–387 (2025). 10.1038/s41565-024-01841-9 39815067 10.1038/s41565-024-01841-9

[108] Z. Li, S. Chu, Y. Zhang, W. Chen, J. Chen et al., Mass transfer printing of metal-halide perovskite films and nanostructures. Adv. Mater. **34**(35), e2203529 (2022). 10.1002/adma.202203529 35908154 10.1002/adma.202203529

[109] J. Shin, H. Kim, S. Sundaram, J. Jeong, B.-I. Park et al., Vertical full-colour micro-LEDs *via* 2D materials-based layer transfer. Nature **614**(7946), 81–87 (2023). 10.1038/s41586-022-05612-1 36725999 10.1038/s41586-022-05612-1

[110] Y. Ren, Z. Wang, Y. Wang, W. Wang, L. Feng et al., Halide perovskite lateral heterostructures for energy routing based photonic applications. Adv. Opt. Mater. **8**(24), 2001347 (2020). 10.1002/adom.202001347

[111] Y.-H. Kim, Y. Zhai, H. Lu, X. Pan, C. Xiao et al., Chiral-induced spin selectivity enables a room-temperature spin light-emitting diode. Science **371**(6534), 1129–1133 (2021). 10.1126/science.abf5291 33707260 10.1126/science.abf5291

[112] P. Odenthal, W. Talmadge, N. Gundlach, R. Wang, C. Zhang et al., Spin-polarized exciton quantum beating in hybrid organic–inorganic perovskites. Nat. Phys. **13**(9), 894–899 (2017). 10.1038/nphys4145

[113] W. Zhao, R. Su, Y. Huang, J. Wu, C.F. Fong et al., Transient circular dichroism and exciton spin dynamics in all-inorganic halide perovskites. Nat. Commun. **11**(1), 5665 (2020). 10.1038/s41467-020-19471-9 33168828 10.1038/s41467-020-19471-9 PMC7653957

[114] Y. Xu, J. Li, W. Xu, X. Fan, S. Yang et al., Elucidating interfacial carrier transfer dynamics for circularly polarized emission in self-assembled perovskite heterostructures. ACS Nano **19**(15), 15030–15039 (2025). 10.1021/acsnano.5c01450 40204749 10.1021/acsnano.5c01450

